# Spirit of the Foreign Periodicals &c.

**Published:** 1840-04-01

**Authors:** 


### Spirit of the Foreign Periodicals.

### On Obstetrical Auscultation.

The following summary of an elaborate thesis, recently published at Strasbourg by Dr. *Carriere*, will probably be read with profit as well as interest.

Dr. C. alludes to the more than ordinary difficulties which attend the successful pursuit of this branch or department of auscultation. He admits that he was often foiled in his early attempts, and that it was only by the most assiduous perseverance that he at length succeeded in being able to recognise the various sounds which have been described as emanating from the uterus when in an impregnated state. He does not however hesitate to assure his readers that any one may, by repeated practice, acquire—provided his hearing is of average delicacy—the necessary aptitude to recognise, and that too with facility, all the auscultatory signs of pregnancy.

Let the young practitioner therefore be not discouraged, if he should fail in his early researches, but let him perseveringly avail himself of every opportunity to accustom his ear to the sounds which are audible in the abdomen, whether in the impregnated or unimpregnated state; and he will probably be rewarded, much sooner than he expects, with success to his desire.

The first point which Dr. C. examines is as to the proper position of the woman during auscultation. Some prefer that she should be standing, others that she should be in the horizontal position. Dr. C. gives the preference to the latter position; although he admits that it is occasionally useful to examine the patient while standing.

The next point is the question of the comparative advantages of the stethoscope and of the naked ear in obstetrical auscultation. There has been considerable difference of opinion here. MM. *Dubois, Hohl,* and *Nægele* give a decided preference to the use of the instrument; while *Ulsamer, Haus,* &c. have rejected it entirely. M. *Stoltz* says that the naked ear suits very well those cases where the abdominal parietes are closely in contact with the uterus, (*immediatement appliquées sur l'uterus,*) and that the stethoscope is preferable in those where it is necessary to press these parts much inwards. On the whole, however, he generally makes use of the instrument. Dr. *Carriere* quite agrees with his preceptor in this opinion. Besides the inconvenience of applying the cheek to the abdomen, the ear, when directly applied, is liable to be confused with the different sounds proceeding from the bowels, and thus is much less fitted to appreciate the strictly uterine *bruits*. Besides, as *Laennec* himself remarked, when we employ immediate auscultation, the physician is obliged to employ more force than when the stethoscope is used in pressing the abdominal parietes inwardly, in order to bring them into close contact with the uterus. This is more especially true in the early stage of pregnancy. Indeed Dr. C. affirms that "it is impossible to auscult with accuracy before the fourth or fifth month unless we use the stethoscope." If the instrument does not render the sounds louder, it certainly makes them clearer and more distinct.

The following is the manner in which Dr. C. proceeds in his examination. He commences by applying the end of the stethoscope, the plug being removed, to the left iliac region, because there the double pulsation or tictac of the fœtal heart is most frequently heard. If it is not audible there, he moves the instrument a little up, then down, then to the outside, and lastly to the inside of the spot from which he started, thus proceeding from the left iliac region as from a centre. As soon as he discovers the pulsation he then follows it step by step as it were, so as to determine the direction in which it is heard, and the precise point at which it is loudest and most distinct.

Even when we discover the cardiac tictac in the left flank, it is always right to examine with equal care the opposite one, as it is possible that another pulsatory sound may be heard on the right side.

Having thus satisfied ourselves of this most important phenomenon, we should then endeavour to detect the other acoustic sign of pregnancy—the blowing or placentary sound.

"In women," says Dr. *Carriere*, "whose pregnancy is but little advanced, as far as the third or fourth month for example, the placentary or blowing sound is to be sought for immediately above the symphysis pubis, the fundus of the uterus scarcely overtopping at this period the plane of the inlet of the pelvis. In such an examination it is quite necessary that the woman should be lying on her back, with the limbs drawn well up so as to have the abdominal muscles well relaxed, and that the stethoscope be used for the purpose of pressing them well in upon the uterus. This is especially important at the early periods of pregnancy; as, then, not only should the abdominal parietes be kept in immediate contact with the uterus, but also this viscus with the contents within its cavity."

Never let the young auscultator abandon the pursuit of this phenomenon, however much he may be foiled in his early attempts. Let him again try the experiments in the same case in the course of a day or two; and let him persevere in this manner, but without fatiguing himself too much at any time, until he succeed in detecting the sought-for sound. *Laennec* has alluded to the intermittence of certain stethoscopic phenomena, and has strenuously urged the importance of frequently resuming the attempt, however unsuccessful the pursuit may be at first.

Dr. C. next alludes to some of the possible causes of error into which the unpractised obstetrical auscultator may fall.

1. The sounds of the heart and of the respiration of the mother are in some cases communicated along the abdomen, and may be distinctly heard, when the ear is applied over the epigastrium. The one sound has been mistaken for that of the fœtal heart, and the other for the placentary bruit. Very slight attention however will guard against such errors.

2. The sounds proceeding from the intestines may disguise, but certainly they cannot imitate, the genuine uterine sounds.

3. Lastly, the sounds proceeding from the muscular contraction of the limbs and body of the observer himself—a sound which resembles the distant rolling of a coach, or the noise of a fall of water—should be guarded against.

On the whole however, it will invariably be found that, when once the ear has been accustomed to the genuine uterine sounds, it is not likely that they will be confounded with any other, as they have a very peculiar and distinguishing character.

We shall now make a few remarks on each of the two uterine sounds.

1. *The Blowing Sound.*—All authors agree that this sound is much more inconstant, fugitive, uncertain, and difficult of detection, than that of the fœtal pulsation. Some indeed have gone so far as to deny its existence altogether; and others have described it in such terms as must lead us to imagine that they in truth never heard it. Dr. *Carriere*, while he admits the frequent difficulty of discovering this sound, assures his readers that its leading characters may generally be detected by any attentive observer, whose ear has been sufficiently practised in obstetrical auscultation. He thus describes it:—

"This phenomenon consists usually in a sort of jerking sound (*bruissement saccadé*) more or less prolonged, and interrupted by regular intervals: it has been frequently compared to the sound proceeding from certain arterial aneurisms.

*Laennec* compares it to the blowing noise frequently heard in the large arteries of chlorotic patients; *Velpeau* to the sound of muscular contraction, or to that produced by strongly compressing arterial trunks. To say the truth, it is certainly not easy to convey to those who have not heard it, a right idea of the uterine blowing sound."

Whatever be its character, it is always strictly isochronous with the pulse of the mother,—becoming duller and more prolonged, when the pulse is slow; and more short and dry, when the pulse is quick. It is never accompanied with any shock; and it is usually limited to a circumscribed spot, decreasing in distinctness as the stethoscope is removed from the point where it is heard loudest. Let it also be remembered that at one moment it may be heard distinctly, and that the next minute it may be quite inaudible; its tone or character being often found to vary in the course of a few minutes. It is generally imagined that the blowing sound is heard on the opposite side of the hypogastrium from that on which the fœtal pulse is perceptible. That this is not always the case will appear from the circumstance that, out of sixty-one cases observed by Dr. *Carriere* and in which the exact position of the two sounds was carefully noted, they—the sounds—were heard on the same side in not fewer than thirty-four.

Of sixty-six cases, where the position of the blowing sound was noted, it was heard on the left side in thirty-eight, on the right side in twenty-eight, eleven times about the middle of the hypogastrium, seventeen times higher up, and thirty-eight times low down; or we may state these results in a different manner, the *bruit de soufflet* was heard twenty-one times on the left side and low down, seventeen times on the right side and low down, eleven times on the left side and high up, five times on the right side and high up, six times on the left side and in the middle, and six times on the right side and in the middle.

With respect to the earliest period at which this sound may be heard, Dr. C. informs us that he has in several cases succeeded in detecting it as early as the end of the third month, when the fundus of the uterus had scarcely passed above the level of the pubis. Far from being diffused at so early a period of pregnancy as *Hohl* has alleged, Dr. C. always found it to be limited to a very circumscribed spot at first.

As this sound is intimately connected with the circulation in the placenta, it is obvious that it must be audible earlier in some cases than in others, as it is well known that the placenta varies a good deal in the place of its attachment to the uterus in different cases; being sometimes near to the fundus and in other cases considerably lower down. The higher up that it is, the more early, we may expect, will be the development of the blowing sound.

As pregnancy advances, it becomes much louder and more readily recogniseable. However it is not always loudest in the eighth and ninth months; being in some cases observed to be much more distinct and obvious in the seventh. The causes of the various modifications of this sound in different cases are certainly not well known.

During the uterine contractions, or *pains*, of labour, the blowing sound becomes much weaker, or is altogether suspended—returning however after the pain has passed away.

It is usually much more distinct after the amniotic waters have been discharged.

Not unfrequently it continues for several hours after the expulsion not only of the fœtus, but of the placenta also. Dr. C. is of opinion that this persistence of the sound after delivery is connected with the more or less imperfect contraction of the uterus upon itself.

Before quitting the subject of the blowing uterine sound, it may be useful once more to repeat that the failure of detecting it on one examination is never sufficient evidence, even with the most experienced auscultator, that it is altogether absent. What may be the cause of its occasional interruptions has not yet been explained; but that such is the case, will not be denied by any who have had ample opportunities of practical information.

It has been alleged, and apparently with reason, that an excessive quantity of amniotic fluid has the effect of obscuring the blowing sound. The attachment of the placenta to the posterior wall of the uterus will no doubt have a similar effect.

Again, the death of the fœtus is probably always followed by a diminution or even by a total suspension of the blowing sound.

As to the proximate cause and seat of the blowing sound, Dr. C. refers them to the circulation of the blood through the placenta.

The opinion maintained by *Haus*, *Bouillaud* and others, that this phenomenon is owing to the compression of the gravid uterus on the iliac arteries, is in his mind altogether untenable. Its seat is by no means in most cases correspondent with the direction of these blood-vessels; and moreover the genuine uterine blowing sound, although analogous to that produced by pressing the end of the stethoscope firmly over the *trajet* of a large artery, is certainly not identical with it.

Dr. *Carriere* has repeated the experiment of *Laennec*, of making the woman lie first on one side and then on the other, so as to cause a variation in the point of the chief compression of the iliac vessels and consequently in the place occupied by the blowing sound—if it be really attributable to such compression; but with no better success than his predecessor.

Without canvassing the opinions of other writers as to the cause of this sound, we shall now briefly state the views of Dr. *Carriere* on this much-disputed point.

"I am quite satisfied in my own mind that the uterine blowing sound has its cause in the passage of the arterial blood in the uterine sinuses, and that in the great majority of cases its seat corresponds exactly with the insertion of the placenta.

In a number of cases where I have passed my hand along the umbilical cord after the delivery of the child, I have always found that the placenta has been attached exactly at that point of the uterus over which the blowing sound had been heard during gestation. In one interesting case of death from convulsions within twenty-four hours after delivery, the place still quite visible on the inner side of the uterus where the placenta had been adherent, was found on dissection to correspond precisely with the spot where the sound had been perceived during life. I believe with M. *Dubois* and others that the mechanism of the blowing sound has something analogous with that of the sound heard over an aneurismal varix—its determining cause being the flux of the arterial blood into the uterine sinuses, a flux which is more active and considerable at the point where the placenta is implanted."

Occasionally, but this is rare, the blowing sound is perceptible over almost the whole anterior surface of the uterus.

"This fact," says Dr. C. "which denotes great activity in the uterine circulation, does not at all militate against the opinion which I profess relative to the relation of the blowing sound to the placenta; for I have never pretended that the dilatation of the uterine vessels was limited abruptly to its circumference; and on the other hand I am convinced that, in those cases where the sound is unusually widely diffused, it is always more decided and sibilant at the one point where the placenta is implanted."

With respect to the value of this blowing sound as a sign of pregnancy, Dr. C. is inclined to consider it as an almost certain indication. He much doubts whether the presence of a tumor in the pelvis or in the uterus itself does ever give rise to the *genuine* blowing sound of pregnancy, although MM. *Velpeau*, *Rayer*, *Bouillaud*, *Bricheteau* and *Stoltz* have asserted that such has been the case.

This opinion of our author is confirmed by the high authority of M. *Dubois* —who denies that the uterine *souffle* is ever produced by any cause independent of pregnancy—and also of Mr. *E. Kennedy*.

With the latter gentleman he also believes that the sound may continue to be heard, but less distinctly, for some time after the death of the fœtus.

The force of the sound cannot indeed be ever taken as a sign of the vigour of the infant; it can only indicate an active circulation in the uterine vessels, and perhaps also the existence of a large placenta.

*The Fœtal Pulse.*—Dr. *Carriere* compares the double *tic-tac* of the fœtal pulse to the ticking of a watch wrapped round with several folds of linen, with this difference only, that the latter sound is drier and of a more metallic sharpness than the former. The number of pulsations varies from 120 to 180; the average frequency may be said to be 140 beats in the minute. In force and distinctness too they are found to differ much not only in different cases, but also in the same case at different times; sometimes they are so distinct that they are at once and without the least difficulty perceptible, whereas in other cases it requires the greatest attention and nicety of observation to detect them at all. Occasionally the fœtal pulsation appears to be simple and not double, as usual, the second sound of the heart's action being quite obscured.

In reference to the comparative constancy of the two uterine sounds—viz. the placentary and the fœtal—it has been asserted by almost every writer on obstetrical auscultation, that the sound of the fœtal heart is greatly more constant than the placentary or blowing noise.

Were we to judge from the results of a single examination in a given number of cases, we should at once assent to such a judgment; but as we have seldom failed ourselves to detect the latter sound, if the auscultation has been repeated several times at short intervals, we are inclined to hesitate somewhat on this point.

"In consulting," continues Dr. C, "a summary of the observations, which I have made since the period when I could place confidence in my auscultatory enquiries, I find that out of fifty-nine cases, the blowing sound was detected in fifty-seven, and the fœtal pulsation in fifty-seven also. In these reports, I find it frequently remarked, 'no blowing sound appreciable;' or, 'blowing sound feeble at such a point;' then afterwards, perhaps in a few days or a week or two, 'blowing sound strong, intense, or even whistling, &c.' It is therefore quite manifest that, before we can establish the relative constancy of the two signs, we must not trust too much to the results of a single, or even of a few observations. If a woman be examined but only once, there is certainly on the whole a greater chance of detecting the fœtal pulse than the blowing sound."

Most obstetrical auscultators agree that the fœtal pulse is rarely, if ever, heard before four months and a half or five months have elapsed. We cannot wonder at this when we remember that the organ, even at this latter period of intro-uterine life, is still very small, and that moreover the fœtus lies low down towards the cervix of the womb, and therefore behind the symphysis pubis. Even for a month or two later, the great relative quantity of the amniotic fluid, the mobility or want of fixed position of the fœtus, the size of which is not yet nearly sufficient to fill the bag of the membranes, &c. are circumstances which tend, even up to the middle of the sixth month of gestation, to occasion many chances of unsuccess.

The sound of the fœtal pulse always becomes louder, and more diffused, when the waters escape, more especially when these have been very copious. The pains of labour have usually the effect of rendering the fœtal pulsation slower, feebler, and less regular than they were before. During a protracted labour, the number of pulsations generally becomes very sensibly diminished not only during the uterine contractions, but also during the intervals of rest.

*Naegele* has found them to fall down as low as sixty-five; and in one case where the umbilical cord was compressed, Dr. *Carriere* found them to be not more than fifty-five in the minute.

Here are the sentiments of our author as to the most common seat of the fœtal pulse.

"In the majority of cases the spot, which corresponds to the greatest intensity of the sound, is situated on a line between the umbilicus and the antero-superior spine of the ileum, or towards one of the iliac fossæ. In some cases the pulsations are found in the line of the *linea alba* between the pubis and the umbilicus; and, in a few rare cases, they have been perceived quite towards the fundus of the uterus.

The extent over which they are audible is very various; being sometimes only two inches square or so, at other times comprising almost the whole of the lumbar and iliac regions of one side; most frequently it is a space which may be covered with the hand."

With respect to the relative frequency of the occurrence of the fœtal pulse in different parts of the hypogastrium, Dr. C. makes the following remarks.

"In eighty-five cases, where I have accurately noted down the exact spot at which the fœtal pulse was perceptible, I found it seventy-four times low down, eleven times high up, forty-five times on the left side, thirty-seven times on the right side, and three times in the middle or along the line of the *linea alba*. Forty-three times its maximum of intensity was low down and on the left side, thirty times on the right side and low down, three times on the left side and high up; and seven times on the right side and high up. I may remark that, in several of the cases where it was heard high up, the pregnancy had not passed beyond the sixth or seventh month."

Before closing our remarks, we shall now briefly allude to one or two other auscultatory phenomena, which are peculiar to pregnancy.

If the fœtus happens to move while the ear is applied over the hypogastrium, we perceive a dull sound accompanied by a pretty brisk shock, not unlike to that produced by striking against a piece of stretched cloth with a smooth rounded and moderately firm body: sometimes it is a sort of undefinable *frolement*, or sensation of grazing sound, attended with a movement which may be felt and often also seen.

This last sound seems to be caused by the rubbing of one or several points of the fœtus against the walls of the uterus. Hence it is not uncommon to find that immediately after its occurrence there is an obvious change in the position, intensity, and frequency of the fœtal pulse, and also in the force and tone of the blowing sound.

The impulse as well as the sound now described are often induced by pressing the extremity of the stethoscope firmly against any point of the hypogastrium. *Naegele* pretends that they may often be perceived, even before the woman is sensible of quickening; but this requires confirmation.—*L'Experience.*

---

### On the Uterine Vagitus of the Fœtus.

Dr. *Marinus*, in a memoir read before the Society of Medical and Physical Sciences at Brussels, has collected together the particulars of a good many cases, in which this occurrence of the infant having been heard to cry before birth has been met with. The following are the circumstances in which this rare phenomenon has been noticed.

1. When the head of the child, the membranes having already burst and the os uteri being well dilated, rests at the inlet of the pelvis, or has descended more or less completely into its cavity, whether it be the face, occiput, or side of the head that presents.

2. When the head is already in the vagina, engaged at the vulva, or fairly protruded beyond it, while the rest of the child is still within the cavity of the uterus.

3. When, in cases of presentation of the feet, knees, or hips, the head remains engaged in the vagina, the body and limbs being already born.

The phenomenon of uterine *vagitus* being once admitted, we are necessarily led to admit that the child can, under certain circumstances, breathe in the uterus. The act of respiration may therefore precede birth; and the child, which has thus breathed, may then die before it is born.

If such may be the case, a powerful argument may be drawn against the conclusions of pulmonary *docimasia,* as it has been called, or the phenomena exhibited by the lungs in cases of suspected infanticide. The objection, however, must not be urged too far; as it is quite true that uterine *vagitus* is a very rare occurrence. The mere possibility, however, of its taking place, justifies and exacts extreme caution in all our decisions, whenever the character and life of a fellow-being are involved.

Dr. *Lados* (Annales de la Societé de Medecine de Gand, 1837,) has related a case, where the *vagitus* was distinctly heard, but the child was born dead: on dissection, the whole of the right lung, and the upper lobe of the left one, were found to be dilated with air; the lower lobe of the left lung being still impervious to it.

The circumstance, therefore, of part only of the lungs being found dilated with air, may possibly be a presumptive post-mortem appearance in favour of intra-uterine respiration having taken place. This opinion will have more weight in any case, if it can be proved that the membranes had been ruptured for a length of time before delivery, and if any manual efforts to complete this had been made by the attendant.

There is still, however, great uncertainty on the whole of this subject; and, as there is a great lack of well-observed and well-authenticated cases on record, we cannot too urgently recommend our medical brethren to examine necroscopically all cases where the intra-uterine vagitus had been heard, and especially those where the children are born dead afterwards.—*Gazette Medicale.*

---

### M. Gendrin's Theory of Menstruation.

The following extract, from the recent systematic work on Practical Medicine, by M. *Gendrin,* will explain his views as to the cause or nature of the menstrual function.

"The observations, which we have presented to our reader's attention, necessarily lead us to modify very essentially the hitherto received opinions on the subject of generation in women. They tend to establish that, during the whole of that period of life when the capability of conception continues, there is a constantly-successive development of vesicles and ovula in the ovaries,—that, at each epoch of menstruation, a vesicle having reached the surface of the ovary becomes the seat or focus of a peculiar organic action, in which all the organs of generation partake,—and that the result of this action is the rupture of the vesicle and the loss of the non-fecundated ovum, either by ovarian destruction or by uterine expulsion.

The recent observations by *Valentin,*—according so well with, and therefore confirming, our deductions drawn from physiological considerations—have shewn that the Graafian vesicles contain or inclose an ovulum, in which are found all the essential parts of a human ovum.

As we find at the same time in the ovarium vesicles in various degrees of development, we cannot well doubt that they exist there only during a limited time or period, from their origin to their spontaneous rupture, which takes place whenever their increase and that of the ovulum are completed. This rupture takes place regularly, at stated intervals, by an organic action, one result of which is the menstrual secretion."

Dr. *Negrier*, professor of midwifery in the Medical School at Angers, has written, we observe, a letter to one of the French periodicals, in which he claims the priority of authorship in respect to the preceding views on menstruation. His words are:—

"The researches of M. *Gendrin* have led him to believe that the menstrual flux is the result of a periodic and regularly-recurring congestion, which takes place every month in the ovaries.

This doctrine I have taught in my lectures ever since the year 1830, and I have repeatedly shewn to my pupils the successive evolutions of the ovarian vesicles from their earliest development to their final rupture, as well as the condition of the uterus at its different phases."— *Gazette Medicale.*

---

### MEMORANDA ON THE DISEASES PREVALENT AT ALGIERS.

The following memoranda are communicated by M. *Guyon*, chief surgeon of the French army in Africa.

"During the last twelve months (1838-39,) *Erysipelas* has been quite epidemic among both the civil and the military inhabitants.

As usual, it has complicated numerous cases of surgical injuries, and has thus added greatly to the general fatality.

The *Small-pox*, too, has been very extensively prevalent among all classes, European as well as African, of the population. Many of the cases—and these sometimes of the most malignant character—occurred in adults who had been vaccinated. During the first six months of 1839, the number of deaths—as well as it could be ascertained—from small-pox amounted to 152. Of these, seven occurred among the soldiers; 35 among the European residents; 78 among the Mussulmen; 28 among the Jews; and four in the Civil Hospital.

The following case of the disease proving fatal on its second attack after vaccination is remarkable.

*Lieutenant Roque*, when admitted into the Hôpital du Dey, presented distinct traces not only of vaccination on his arm, but of a previous attack of variola on most parts of the face and body. He had been a pupil of the Ecole Militaire at Paris, into which, it was well known, no one who has not been vaccinated can be received. The disease, variola, with which he was affected at his admission into the hospital, speedily assumed a malignant character, and it proved fatal in the course of a few days.

M. *Guyon* adds, that cases of small-pox occurring twice in the same individual are not very unfrequent, and reports the following interesting example from his experience.

The late Baroness *Tascher* used to mention, that among the officers of the garrison in the island of Martinique,* who were in the habit of visiting at her father's house, there was one who, being very much disfigured by the marks of the small-pox, was good-humouredly bantered by his messmates that

* The arrival of a slave ship in any of the West India islands used to be very frequently followed by the breaking out of numerous cases of small-pox.

he was surely proof against the return of the enemy. During an epidemic prevalence of the disease he was, however, again seized with it, and he died of its effects.

*Tetanus*—the spontaneous as well as the traumatic form—prevails sometimes almost as an epidemic. Three cases of the spontaneous disease occurred at Oran in the beginning of April, when the heat suddenly became very great. They were all treated with general and local bleeding, cupping along the spine, internal use of large doses of opium, and hot baths: two proved fatal.

In the following month, three cases of traumatic tetanus occurred: in one of these, it followed upon the insertion of a seton in the neck.

It is well worthy of notice here that the disease was *epizootic*, at the same time, among the horses and mules of the garrison. One of our veterinary surgeons had seen nine of these animals affected with tetanus: all of them died.

At Bona, four cases of spontaneous tetanus occurred during the first three months of the year—one only of the patients survived.

M. *Guyon* is of opinion that the sudden transitions of temperature are among the most powerful causes of the disease. It is to this influence that we may probably ascribe the great number of cases, which occurred among the troops during the expedition to Constantine in 1836, and our subsequent retreat to Bona. The majority of those who suffered amputation died from tetanus supervening.

But a much larger proportion of the tetanic patients had suffered no wound at all: the disease was of spontaneous origin, and in them, it was doubtless attributable to the great change of climate from the severe cold which we had experienced under the walls of Constantine, to the high heat when we returned to Bona.

M. *Guyon* accounts for the frequency of the disease among the negroes, more especially among their infants, in the West Indies, by the rapid and great variations of temperature to which they are exposed from being badly lodged and clothed.

The custom of the natives of hot climates anointing their bodies with some unctuous substance has no doubt the effect of rendering them less sensitive to the injurious influence of sudden transitions from heat to cold.

Among the soldiers who were attacked with tetanus, during and after the expedition to Constantine, there were two in whom the disease did not manifest itself till nearly two months afterwards. In both cases too the patients were, at the date of their seizure, far removed from the place where, so to speak, they had caught the germ of the disease. One had been admitted into the hospital of the Dey at Algiers with a *gastro-bronchite*; he was suddenly seized with tetanus and died in twenty-four hours. The other poor fellow had suffered amputation of both legs at Toulon, in consequence of mortification induced by the severe cold during the expedition; and he died on the following day, the tetanic symptoms having set in about twelve hours after the operation.*

Diarrhœa, dysentery, and bronchitic affections are of frequent and fatal occurrence in our possessions on the African Coast, more especially at Oran and Constantine—these two places being more exposed, and therefore being more subject to rapid variations of climate.—*Gazette Medicale.*

---

* M. *Guyon* remarks that in the greater number of the cases where tetanus had supervened after amputation and proved fatal, the operation had been performed for gangrene of the limbs induced by cold, and not for severe gun-shot wounds.

### ON A PECULIAR TREMOR OF THE FINGERS IN WRITING.

The following extract is taken from a recent Number of one of the German Journals. The complaint of which it treats is rare, and has not been described in any systematic work on medicine that we know. It consists in a tremor or involuntary shaking of the fingers of the right hand, whenever the patient takes hold of a pen or pencil for the purpose of writing or drawing. What is very strange is that some patients can mend a pen, shave themselves, or play on a musical instrument without difficulty or any unsteadiness of their hand; but no sooner do they touch the writing paper with a pen, than they are immediately seized with a trembling of the fingers, so that perhaps they cannot trace a single legible word. When they cease to write the trembling ceases.

In *Kopp's* patient, the trembling of the thumb and fore-finger was accompanied with a most unpleasant and almost painful sensation in the affected parts, which gradually became more distressing if the patient persisted in attempting to write, and ultimately forced him to lay the pen down. This sensation was often experienced in the palm of the affected hand, even when he was not writing. *Gierl* mentions that his patient experienced on any attempt to use his pen rapid contractions of the thumb, fore and middle fingers, and a peculiar feeling of compression or constriction, as if a metallic plate was fixed around the wrist. If he continued writing, the tremor of the flexor and extensor muscles extended along the whole arm up to the axilla; and this was so decided that it was manifest to the eye. At the same time, he complained of a feeling of pressure on the outer side of the arm, at the place where the deltoid muscle is inserted into the humerus; the other muscles remained in repose, unless indeed the patient still persisted in attempting to write. If this was the case, a feeling of faintishness and of general malaise came on, and the body was bathed in a profuse perspiration. Whenever the pen was laid down, all the uneasiness at once vanished. This patient never experienced the slightest difficulty either in lifting the heaviest weights, or in performing the most delicate mechanical operations.

This trembling of the fingers usually comes on very gradually and almost imperceptibly. At first, the patient experiences only a very slight inconvenience after writing long. By degrees it increases, so that he is obliged often to leave off in the middle of a page; and, in spite of all his efforts, he cannot go on. The complaint seems to be quite local, the general health being usually quite good all the time. Strong mental emotions, the use of spirituous drinks, seminal emissions, &c. seem to aggravate it—in short, whatever renders the system more irritable and sensitive is sure to increase the local annoyance.

It is not easy to suggest any remedy for this troublesome affection. Some patients have found advantage from wrapping something round the pen, so as to make it more bulky; others from fixing it to the fore-finger by means of a metallic ring, and others again by applying along the inner side of the hand a piece of wood, so as to prevent the fingers from being drawn in towards the palm. A certain relief is sometimes experienced by placing the pen between the fore and middle fingers, or between the middle and ring fingers.—*Gazette Medicale.*

*Remarks.*—We have frequently experienced something like this spasmodic trembling of the fingers during writing. In our own case, it has never amounted to an actual tremor of the muscles, but has been limited to an annoying sensitiveness at the points of the thumb and forefinger, and to a sense of uneasiness and constriction at the metacarpal joint of the former. We are not aware of any particular cause which has tended to bring it on. When once present, the circumstance of the attention being directed to the part, has always the effect of aggravating the annoyance, so that the only plan to get rid of it is to cease writing at the time, and occupy the mind with something to divert the attention.

*Hunter*, or some other celebrated man, remarked that, by having his attention directed for a length of time to any part of his body, he could actually induce a painful feeling in that part.

The remark is quite true; and our own experience at this very moment illustrates its truth. For already, at the present moment, have we begun to feel the old annoying tenderness in the point of our writing thumb, and the wrist and joint of the thumb are beginning to be excessively weary, although we have not held the pen long.*

Whoever has seen much of nervous diseases, well knows the importance of diverting or withdrawing the attention of the invalid from the seat of his or her distress.

---

### Discussion respecting the Phrenological Organ of Language.

M. *Bouillaud*, at a recent sitting of the Royal Academy of Medicine, introduced the subject of the seat or locality of the Phrenological Organ of Language, and narrated several cases in confirmation of *Gall* and *Spurzheim's* opinion, that it is placed in that portion of the anterior lobes which rests on the roof of the orbits.

In a previous memoir, which M. *Bouillaud* communicated nearly 15 years ago, he had collected the details of 64 cases, to shew that this organ is seated in the anterior lobes of the cerebrum, as first announced by Gall and confirmed by the observations of Spurzheim. On the present occasion, he added the reports of thirteen other cases, which have come under his notice, and all of which, he thinks, tend to prove the correctness of the phrenological doctrine. He admits that several instances, apparently opposed to this doctrine, have been published by MM. *Cruveilhier, Andral, Lallemand,* and others; but he insists that the details of these cases have been always unsatisfactory, and never sufficiently complete for the purposes of an absolute decision.

M. *Rochoux* was the first to reply to the observations of M. *Bouillaud.* "Numerous facts," said he, "have abundantly proved that the faculty or power of speech has remained intact, when the anterior lobes of the brain have been seriously injured or diseased; and, on the other hand, that this faculty has been lost, when no discoverable lesion of these parts has been afterwards found. I am ready to admit that a number of cases may be adduced, which seem at first sight to be directly favourable to M. *Bouillaud's* conclusions; but then there are a great number of exceptional cases, which are quite opposed to them; and we must ever bear in mind that one absolutely authentic negative case is sufficient to upset the authority of a hundred seemingly affirmative cases. In that form of paralysis, which occurs in the insane, the loss, or at least, a lesion of the speech is usually one of the earliest symptoms. It is especially on the muscles of the tongue that the paralysis is at first directed; and yet in such cases we have no reason to believe that the anterior lobes are more affected than the other parts of the brain;† as the morbid change seems rather to involve the whole surface of the organ."

---

* A partial relief is obtained by holding the pen between the forefinger and thumb, and thus removing its pressure entirely from the tip of the latter.

† This objection is certainly not a valid one, and, when examined attentively, will be found to arise from a mistaken view of the subject. The phrenological

M. *Cruveilhier* followed M. *Rochoux.* He maintained that lesions of speech are not more necessarily or more frequently connected with injuries of one than of any other part of the brain; and that they are as common after injuries and diseases of the posterior as of the anterior lobes.

He repeated the assertion of M. *Rochoux,* that this faculty may be lost, although the anterior lobes are intact; and again, that these lobes may be deeply diseased, the faculty remaining entire.

" It is quite right, continued he, " that we should remember that there are three distinct elements, so to speak, involved in the phrenological faculty of language:—1, there is the memory or recollection of things; 2, there is the memory of words; and 3, lastly, there is the power of articulating sounds, or, in other words, of speech.

When the memory of things is impaired—which often happens after external injuries of the head, malignant fevers, &c.—the patient is usually in a state of imbecility, all the intellectual faculties being more or less stupefied or perverted: this is more frequent than the isolated loss of the memory of words. Words are most intimately, or rather necessarily associated with ideas. We can scarcely have an idea without having some corresponding sign or symbol of it; but as this union or association is altogether conventional, it may not exist; and thus the outward symbol may be lost, or awanting to the idea. . . . . . Again, the memory of ideas and of words may be preserved, while the movements of the tongue requisite for speech, as well as for taste and deglutition, may be lost.

The patient may comprehend the questions put to him; he may remember what he has seen and heard; but perhaps he cannot articulate a syllable. Every medical man is acquainted with such cases; in one, which recently occurred in my practice, a woman had quite lost the power of articulating the name of her husband, although she retained a perfect recollection not only of things, but also of all other words.

As to the seat or locality of the cerebral lesion in such cases, I admit that it is frequently in the anterior cerebral lobes; but then this coincidence is by no means uniform and constant, as is alleged by M. *Bouillaud.* I could adduce numerous cases of lesion of these lobes, in which there was no decided affection of the speech. As to the cases in which the anterior lobes have been found deeply disorganised, or even altogether wanting, several have occurred in my practice; but M. *Bouillaud* will perhaps say that such cases are by no means so conclusive as at first sight they may appear to be, since the morbid change was limited to one hemisphere of the brain. If he insists upon the force of this objection, then we must avail ourselves of it in reference to the cases which M. *Bouillaud* himself has reported in illustration of his views, where a lesion of one only of the anterior lobes was accompanied with a lesion of the alleged function. I have related in my Pathological Anatomy the case of an idiot, in whom both anterior lobes of the brain were wanting, and who yet exhibited no alteration in the faculty of language.*

---

faculty of language is certainly not the same as the faculty of speech or articulation. One is, in truth, the memory of words; the other is the mere power of expressing or enouncing them.

The faculty of language may be perfect, although the speech may be more or less injured; just in the same manner as a musical performer's talents may remain perfect, although they can no longer be displayed, in consequence of some injury to his instrument.

* There is surely self-contradiction here. Does an idiot ever possess a correct memory of things and of words, as well as the power of articulation—the three elements which M. Cruveilhier himself admits to be comprised in the faculty of language?

I could easily prove that almost every cerebral lesion, of whatever part of the organ, may induce an alteration in the faculty of speech. Such an alteration is, it is well known, one of the earliest signs or symptoms of hemiplegia.

In a case of this sort recently under my care, where the speech was entirely lost, but the intellect remained clear and unaffected, the *tuber annulare* only was found, upon dissection, to be diseased, the anterior lobe being perfectly sound. (Does not this very case militate against M. *Cruveilhier's* and in favour of M. *Bouillaud's* views? The memory of sounds and words remained, and the anterior lobes were healthy; the power of speech only was gone, and the *tuber annulare* situated above the origin of the hypoglossal nerves was diseased.)"

In conclusion, M. *Cruveillier* infers:—

1. That it has not been shown that the faculty of language has a special seat in any particular part of the cerebral mass, and still less that the seat is in the anterior lobes.

2. That every deep and serious morbid change of the brain, whatever be the part affected, is usually accompanied with some lesion of speech.

3. That the plurality of cerebral organs cannot be demonstrated.

M. *Blandin* followed; he related the following case in confirmation of the phrenological doctrine.

A child received a musket-ball in the orbit; after destroying the eye it traversed the exterior orbitar plate, and made its escape just in front of the ear. This child, whom I saw at the Hôtel Dieu several weeks after the accident had lost all power of articulation. During its recovery, it was necessary to teach and accustom it to pronounce words. When it left the hospital, it was able to articulate its own name, and a few other words. Now in this case is it not highly probable that the superior orbitar plate was fractured, and that an inflammatory process had extended to the point of the anterior lobe which rests upon it?

As to the objected cases, in which the lesion of other parts of the brain seemed to occasion a loss or some disturbance of speech, they prove nothing in my opinion against the opinion of M. *Bouillaud;* for the corpus striatum and the thalami optici, for example, contribute by their irradiations to the formation of the anterior lobes; and the same may perhaps be said of the fibres of the upper part of the medulla spinalis, which are in truth the primary roots of these parts.

On the other hand, even if it can be shown that in some cases, where there has been disease of the anterior lobes, the speech has not suffered, it may possibly be that the immediate seat *du principe coordinateur* was not involved in the lesion. M. *Bouillaud* has not affirmed that the whole of the anterior lobe presides over the faculty of language; a small portion of it only being, according to the phrenological views, the seat of this power.

M. *Martin Solon* adduced the following cases from his own practice in confirmation of the same doctrine.

1. A girl had been for a length of time affected with slight impediment in her speech; gradually she lost the power of uttering even a syllable, and every movement of the tongue was lost. Symptoms of contraction and spasm came on, and she died. On dissection, besides the usual traces of chronic meningitis, several hydatids were found in the medullary substance of the anterior cerebral lobes.

2. In another case, whose progress and general character had been very similar to those in the preceding one, there was found on dissection a well marked softening or *ramollissement* of the anterior lobes.

3. A young girl lost her speech after a severe attack of fever. When all the active symptoms had disappeared, frictions over the forehead with an ammoniated pommade were employed and the speech gradually returned. Similar success attended the use of the same means, in a case where the loss of speech followed a suppression of the catamenia.

M. *Gerdy* expressed himself unsatisfied with such cases as those adduced by the preceding speaker.

One single well-authenticated case of decided lesion of the anterior lobes, in which the speech was unaffected, is quite sufficient he thought to overthrow the phrenological doctrine. Now many more than one such case may be adduced. Thus, in the remarkable instance reported by M. *Andral*, of a youth who suddenly lost his speech, sight, and intellect, all the symptoms disappeared after a certain time; the speech returned, and also the intellect and vision. Subsequently this youth died—the amendment having however continued to the last,—and on dissection the right anterior lobe of the brain was found to be excessively softened. M. *Bouillaud* himself is candid enough to acknowledge that it is a very curious instance, and not easily explicable. Many other such cases are to be found in various pathological records.

M. *Ferrus*, one of the physicians of the Bicetre Hospital, replied to the observations of M. *Gerdy*. He was quite willing to admit that, while avowing his belief in the truth of the general principles of phrenology, he did not give his assent to every doctrine inculcated by its professors. He thought that it required many restrictions, and stood in need of not a few modifications; but in his opinion it had already thrown too valuable a light upon the functions of the brain to merit such reproaches as had been used by MM. *Cruveilhier, Gerdy, Rochoux, &c. &c.* He had satisfied himself by numerous researches that the faculty of language is really seated, or, in other words, has its material organ in the convolutions of the anterior lobes. I have, says he, over and over again observed that in those persons, who are gifted with a striking memory of words and an aptitude for the acquisition of languages, the eyes are usually prominent and full, in consequence of the great development of the inferior portion of the anterior cerebral lobes. The lower part of the forehead too is usually projecting in such persons.

My pathological observations have led to a similar result; and they are certainly not at all in accordance with those of M. *Rochoux*. I have, for example, repeatedly observed that, in the majority of cases where the speech had become affected after the delirium of insanity, the anterior and inferior convolutions of the brain exhibited striking alterations, either as to the consistence of their cineritious matter, or in the relations between it and its investing membranes, or in the more deeply-seated medullary substance. Three days ago I examined the body of an insane, almost idiotic, patient, who had become paralytic: for a length of time his speech had been confused, and his condition presented no chance of amendment. More lately his symptoms had become more acute and violent. The excessively congested state of the blood-vessels of the meninges and of the substance of the brain accounted for this aggravation; while the unusually strong adherence of the pia mater to the special convolutions—designated by *Gall* as the organ of language—afforded a rational explanation of the loss of speech in this case.

M. *Ferrus* adduced two or three other similar examples in confirmation of the phrenological doctrine on this subject. He then alluded to the cases reported by *Andral* and others, which appear to be conflicting with these; and suggested that most of them are far from being conclusive, seeing that in by far the larger majority one organ only of the faculty has been injured, its fellow in the other hemisphere remaining the while almost or altogether intact. But even admitting that both organs have been found seriously altered, without the faculty being deeply disturbed, such cases by no means afford so decisive an argument against M. *Bouillaud's* views as some of my learned colleagues appear to imagine. Every pathologist knows how frequently different parts and viscera are found after death almost totally disorganised, which had given out during life no very decided symptoms of disturbance or disease. If this be true of the brain *en masse*, and of other organs, why may it not be equally so of parts of the brain?

M. *Bouillaud* closed the discussion—which had continued during three *seances* of the Academy—by replying to the various arguments and facts which had been adduced by his opponents.

Certainly we must acknowledge that the phrenological doctrine has on the whole very successfully resisted the attacks of its adversaries during this rather protracted ordeal, and that not a little merit is due to M. *Bouillaud* for his manly and able defence of his positions. Even M. *Cruveilhier* admitted that he did not combat *à priori* the localization of the faculties of the mind; moreover, that he willingly acknowledged the utility of phrenology, and the services which it had already conferred; "but," continued he, "I have not yet heard sufficient reasons to place the organ of language in one part of the brain to the exclusion of other parts.—*Gazette Medicale.*

---

PROSECUTION OF A PATIENT BY HER DOCTOR IN FRANCE.

The following curious case affecting *doctors* was recently decided by the Court of Cassation, the highest judicial tribunal, we believe, in France. It appears that Professor *Mojon* of Geneva had in 1833 engaged to give his professional services to the *Baroness Feucheres* and her household, during his entire life, on the express condition that she agreed to assure *à perpetuité* to him, his children, and their descendants in a direct line, an annual sum of 10,000 francs—with the permission to her of at any time annulling it by a payment of 200,000 francs at once.

M. *Mojon* had in consequence been induced to quit Geneva, and to take up his residence at Paris. The parties continued to fulfil their mutual engagements up to 1837, at which period the *Baroness,* without assigning any reasons, thought proper to *break* with the Doctor.

He cited her forthwith before the *Tribunal de Premiere Instance,* who at once decided in his favour. The Baroness thereupon appealed to the *Cour Royale de Paris* against this judgement; but again she failed; not only the judgment of the lower court being confirmed, but all expenses being allowed to Dr. M.

The lady however was resolved to try the chance of law once more. So she carried the case before the highest tribunal, in the hope of obtaining the *cassation* or reversal of the verdict. The grounds of this appeal were that the engagement, into which Dr. *Mojon* had entered, involved a violation of the 1131, 1133, and 1780 articles of the civil code, in which it is expressly laid down, "it is forbidden to any one to contract an obligation by which his independence and liberty are alienated."

The Advocate General, who was employed by Dr. M. as his counsel, very ably argued that such a prohibition could not possibly be shewn to extend to such an agreement as that which had been entered into between his client and the defendant. The article 1780, which forbids any one to engage their services for life, is applicable only to workmen and domestics, and certainly not to the members of so honorable a profession as that of medicine.

Again the article 1781, which lays down the obligations of a *master* in reference to the payment of wages or salary, cannot surely be made to embrace the claims of a physician or surgeon for their professional services: for how can any one, who employs a medical man, be said to be his *master?*

For these and many other similar reasons, the appeal of the Baroness was again rejected; the contract between her and Dr. M. was confirmed; and she was condemned to pay all expenses.

We have heard that the Baroness has subsequently summoned Professor Mojon *pour lui donner les soins assidus qu'exige l'etat de sa santé.—Gazette Medicale.*

### ON THE PATHOLOGY OF THE VEINS OF THE ABDOMEN.

Although frequent allusion is made, in the writings of former physicians, to the important influence of the abdominal venous system on various diseases, yet very little notice has hitherto been taken of its pathological alterations. *Bichat*, with the view of shewing that the blood itself may become diseased, has recorded in his General Anatomy a single case, where on dissection he found the vena portæ and its principal branches filled with a sanious fluid.

It is to M. *Bouillaud* that we are indebted for the first accurate information of the relation between obstructions of the *portal* system of veins and ascites. MM. *Reynaud* and *Cruveilhier* have since contributed some useful observations on this pathological question.

M. *Defresne*, a physician in Paris, is about to publish a work on the diseases of the liver and the pancreas, wherein he has examined at considerable length the morbid changes to which the abdominal veins are subject. He has communicated his observations to the *Gazette Medicale*, from whose pages we have drawn the following remarks.

The changes most frequently observed in this system of veins are the result of an inflammatory action induced on their internal tunics in consequence—at least generally—of the absorption of acrid matters from the bowels into their cavities. Hence the chief morbid appearances consist in redness, softening, rugosity of, and deposition of lymph on, the lining membrane of the affected veins.

These vessels sometimes become embossed or partially varicose. Their contents are always more or less altered, from simple coagulation of the blood to an admixture with or a total substitution for it of purulent matter. This matter is sometimes thick and creamy; at other times it is sanious and fetid. The mesenteric, the splenic, and the hepatic veins have, in different instances, been found completely obstructed from coagula within their tubes. The portion of the veins immediately below the seat of the obstruction is usually much distended and varicose. In one case, where M. *Jobert* found one of the mesenteric veins quite plugged up, the rectal veins were enormously dilated. It has with some reason been conjectured that engorgement of the spleen is often owing to obstruction of the splenic vein.

These various morbid appearances have been more frequently observed in the hepatic veins and in the branches of the vena portæ than in any other. Purulent matter has been found in these vessels, extending from the larger even to the minutest ramifications. M. *Cruveilhier* describes the appearances he noticed in one case in the following words:—"There was in the transverse fissure of the liver a purulent deposit, which surrounded the trunk of the vena portæ: from the abscess, as from a centre, numerous purulent canals proceeded out to the divisions of the vein, following the course of these vessels most exactly. The capsule of Glisson was much thickened."

The parenchymatous substance of the liver around and adjacent to the diseased veins is always more or less altered from its healthy appearances: it is usually darker coloured, softer, and more friable.

In some cases the vena portæ has been found almost, or altogether, obstructed. The manner, in which the abdominal circulation is then carried on, is described by Dr. *Dufresne* as follows:

"The first change is the dilatation of the vein immediately below or behind the point of obstruction. In proportion as the obstruction becomes more complete, the various collateral and anastomosing communications between the different branches of the portal system and the systemic system of veins become more and more enlarged. The blood of the abdominal system, which formerly returned to the heart only after having traversed the liver, passes now at once by collateral branches into the general venous system. All the mesenteric veins become enormously dilated. The capillary veins of the thorax and abdomen are in most cases also much enlarged; and this enlargement may extend even to the veins of the viscera within. The surface of the intestines and diaphragm, in particular, has sometimes presented a net-work of highly injected vessels. The intercostal, diaphragmatic, and azygos veins have been in several cases found greatly distended. These vessels will be enlarged still more, if there is any obstruction of the vena cava itself at the same time. In one case, related by Dr. *Baillie*, this venous trunk was found obliterated at the point where the hepatic veins join it. The blood of the general and of the abdominal systems had reached the heart chiefly through the two azygos veins, and the anastomoses between the lumbar and the intercostal veins.

Without dwelling longer on the pathology of the subject, let us now proceed to say something of the *causes of inflammation of the abdominal venous system.* Severe contusions of the abdomen and loins have seemed to have occasionally given rise to it. The pressure too of tumors of any sort within the abdominal cavity has had the same effect. Probably however by far the most frequent cause is the direct absorption of acrid matters from the inner surface of the bowels. In more than one instance, the inflammation of the vena portæ, or of some of its branches, has been found co-existing with irritation or inflammation of the digestive passages. MM. *Ribes, Andral*, and *Bouillaud* have reported several cases which distinctly prove that a phlegmasia of the intestinal mucous surface may extend along the mesenteric veins of the vena portæ.

Inflammation of these veins has been observed after fatal hernia, prolapsus recti, fistula ani, cancerous and other forms of ulceration of the bowels, and in numerous cases also of typhus fever. In some examples of malignant disease, cancerous or encephaloid matter has been discovered within the diseased vessels.

It is not easy to give any accurate account of the *symptoms*, which accompany a diseased state of the veins of the abdomen. We may expect indeed that there should be a general feverishness, occasional shiverings, nausea and vomiting, perhaps diarrhœa, a clayey or an icteric colour of the skin, pain or tenderness of the abdomen, loss of appetite and great prostration of the whole system. If the disease lasts for some time, emaciation and marasmus will necessarily supervene. The feverish symptoms sometimes assume an intermittent character; the paroxysm usually returning every day. If the patient has been subject to piles, these are always, as indeed we might expect, much aggravated—in consequence of the accumulation of blood in the mesenteric veins. In one or two cases of obstructed vena portæ, the superficial veins of the abdominal parietes have been observed unusually distended and almost varicose. In other cases, however, this appearance has not been noticed.

M. *Duplay* gives the following description of a case, where the vena cava and the right branch of the vena portæ were found on dissection to be quite obliterated.

"From each groin there proceeded a vein, the epigastric, as large as the little finger: as it mounted upwards, it was divided into a number of considerable branches, which anastomosed freely with each other, and also with their fellows on the opposite side, so that the front of the abdomen was covered with a network of vessels, from which numerous smaller ones proceeded, some passing along the intercostal spaces, and others as high as the arm-pits. Around the knee and under the skin of the legs were observed a multitude of veins, such as we frequently notice in some women during pregnancy."

The last symptom of a diseased condition of the internal abdominal veins that we shall mention is of more constant occurrence than any to which we have as yet alluded—viz. the ascites, or dropsical effusion of the abdomen, which almost always accompanies the obliteration of the vena portæ or any of its large branches.

It is to M. *Bouillaud*, as already noticed, that we owe the first precise information on this interesting subject of pathology. His memoir will be found in the *Archives Generales* for 1823.—*Gazette Medicale.*

---

### TREATMENT OF MALIGNANT ULCERS WITH CORROSIVE SUBLIMATE EXTERNALLY.

"I have been engaged for the last fifteen years," says Dr. *Ordinaire,* "in testing the value of the corrosive sublimate as a caustic in the cure of ulcers of malignant character, and in the removal of cysts and other tumors. My mode of employing it is very simple; the ulcerated surface is covered or coated with a pinch of the sublimate in fine powder: the quantity never exceeding seven or eight grains. A piece of diachylon plaster is then laid over all. When the caustic is applied to any inward surface, as to the mouth, rectum, &c., some lint or carded wool should be placed upon it, in order to prevent it from extending to the neighbouring part.

No sooner is the sublimate applied to a wound than an intense burning pain is caused; this usually lasts for several hours, and is usually attended with great tumefaction of the surrounding parts. The use of emollients, and of leeches if necessary, will however speedily relieve these symptoms.

In cases of superficial cancerous ulcerations, one application of the caustic is sometimes sufficient; but generally it is necessary to repeat it. The eschar however produced by the first must have either fallen off, or been removed by scissors or the knife, before the sublimate is re-applied. If, after the second or third application, the granulations are fungous or otherwise unhealthy, we should not hesitate to destroy them by again covering them with caustic powder. The healing of the sore left by the falling off of the eschar is generally remarkably rapid."

The following cases, extracted from a multitude recorded in his notes, are given by the author in illustration of the preceding remarks.

*Case* 1.—A woman, 50 years of age, consulted Dr. *Ordinaire* for an ulcerated fungoid tumor situated in the mouth upon the right maxillary bone. It had existed for upwards of a twelvemonth. The original tumor had been excised, and the surface been cauterized with a hot iron; and, as the disease returned, she was advised to submit to the removal of a portion of the superior maxilla.

The fungus, at the time that Dr. *Ordinaire* first saw the patient, was of the size of a kidney-bean, and occupied the space of the canine and lesser molar teeth,—which had been extracted—extending upwards along the protuberance of the upper jaw. The fungus frequently bled, and was the seat of severe lancinating pains.

Dr. O. after removing all the diseased parts by means of scissors, covered the wound with the corrosive sublimate powder; this he repeated several times until its surface was invested with a thick white crust or layer. Some carded cotton was then laid upon it, in order that the opposite surfaces of the cheek might be protected from the caustic. On the next day, without waiting for the falling off of the eschar, it was removed by means of small scissors, and the sublimate was re-applied. After the third application, the lancinating pains in the cheek had very sensibly diminished in severity. Dr. O. adds, "I thus followed the fingers even into the maxillary sinus, (!) and I obtained a perfect cure after the sixth application."

Two years afterwards, this patient again consulted Dr. *Ordinaire* for a fungous tumor situated below the right clavicle, and which, within a very short time, had attained the size of a filbert. One pinch of the sublimate powder sufficed to destroy it completely.

*Case* 2.—A gentleman, 74 years of age, had a cancerous ulcer in his lower lip, which had during two years resisted frequent cauterizations practised by an empiric, whom he had consulted.

He had been advised to allow a portion of the lip to be excised, when he accidentally heard that Dr. O.—to use the Doctor's own words—possessed a means of curing cancer. The sublimate was freely applied over the ulcerated surface, so that it was coated with a tolerably thick layer of the powder: it was then covered with simple dressing.

The pain was very severe at the time; and great swelling of the entire lip supervened. This however subsided in 24 hours, under the use of emollient cataplasms. On the fourth day a thick eschar was detached, and at the end of the third week the cicatrization of the wound was complete. The caustic had not been applied a second time.

*Case* 3.—A man, aged 32 years, had for many years been subject to a *prolapsus recti.* For some time before he consulted Dr. O. the gut had not been replaced; and the protruded portion had a most unhealthy appearance. It was swollen and furrowed with ulcerated lines, from which oozed a sanious discharge. The patient experienced severe lancinating pains in the part. "Recognising," says Dr. O. "a cancerous degeneration, I at once removed with the knife the whole of the tumor which protruded beyond the margin of the anus, and, being assured in my own mind that there were some unhealthy excrescences within the gut, I determined to destroy them with the sublimate. I prepared a tampon or bougie of lint, smeared it well with lard, and then powdered it over with the sublimate: this I introduced to two inches depth into the rectum. The patient immediately experienced most intense pain; and it was with great difficulty that Dr. O. persuaded him to bear the bougie for a few hours. On the fourth day thick eschars separated by the use of injections and of hip-baths; and no fresh applications of the sublimate were made, except to one or two suspicious-looking points at the margin of the anus.

Thirty-two days after the first cauterization, the patient was entirely well. For twelve years afterwards he continued to enjoy perfect health.—*Gaz. Med.*

*Remarks.*—We are unwilling to make any remarks on the preceding cases, further than to warn our younger readers from being too bold in the use of so severe a remedy as the one proposed, especially in cases of *actually* malignant disease. That wonderful cures however have sometimes been effected by the use of the corrosive sublimate as a caustic is known to most experienced surgeons. Dr. *Ordinaire* seems to be a regularly educated and a scientific surgeon, for we observe in a recent number of the *Gazette* he has published one or two cases of strangulated hernia, where he performed the operation of dividing the stricture, without opening the sac, with perfect success.—*Rev.*

---

### On the Use of the Starched Bandage.

Our readers are no doubt well aware that, for some years past, the use of what has been called the *bandage amidonné*—which is nothing but a calico bandage or roller wetted in a strong solution of starch and then applied in the usual manner round a limb—has been extensively adopted on the Continent in the treatment of fractures, more especially in military practice. It is to M. *Seutin*, one of the leading surgeons of the Belgian army, that we chiefly owe its recent introduction. Whether there is any truth in its having been used by *Cheselden*—as the national pride of England, M. *Seutin* says, has thought fit to assert—we really do not know. But even though there be, there cannot be a doubt that our author deserves well of his professional brethren by his able and zealous recommendation of the practice. Already has it been extensively adopted in France and Belgium; and so highly is it thought of in Russia that all the military and naval surgeons there have been ordered to be instructed in its use and to adopt it in their practice.

M. *Seutin* has recently addressed a letter to the Royal Academy at Paris, with the view of pointing out the various surgical maladies in which he has used his *bandage amidonné* with useful effects. He instances false articulations, section of tendons, sprains and luxations, white-swellings and other tumors, spontaneous dislocations, necrosis and caries of bones, and various affections of the joints. To these he adds herniæ in young children, varices, articular rheumatism, the tremor of stumps after amputation, and after the excision of the diseased parts in such cases as cancer of the lip, &c.

The following are the advantages enumerated by M. *Seutin* of the *bandage amidonné* in the treatment of fractures.

1. It is at the same time a removeable and an unremoveable apparatus.
2. It exerts a circular compression round the whole limb, by which a more exact apposition of the fragments is maintained than by other means.
3. It enables the patient to move about with the assistance of crutches.
4. It allows a free exit to the purulent discharges in cases of compound fractures.
5. It is light and not cumbersome, and applies itself closely to the limb.
6. It is easily cut and taken off, when its removal is necessary.
7. Its materials are cheap and are to be had every where: hence it is exceedingly well adapted for military service.
8. By merely moistening it and squeezing it between the fingers, it may be adjusted, after it has been once applied, at any particular point where it may happen to lie unsmoothly and cause undue constriction.
9. In some cases where it is desirable to have it as soft and pliant as possible it will be useful not to moisten with the starch mucilage the internal surface of the bandage.
10. It permits any position of the limb, either of flexion or of extension, and also the movements of the joints towards the close of the treatment, for the purpose of preventing anchylosis.—*Gazette Medicale.*

---

### REMARKS ON M. GUERIN'S RECENT PROPOSAL.

M. *Bouvier,* who is a well-known *Orthopædist* of the French Metropolis, has addressed some remarks on the rather strange proposal which his rival M. *Guerin* has recently made to divide certain muscles of the back, for the cure of lateral deviations of the spine. M. B. denies that the contraction of the spinal muscles plays the chief part in the production of these deformities, as alleged by M. Guerin. His dissent rests on the following grounds:

1. When an attempt is made in the living subject to *redress* a lateral curvature of the spine, we do not observe that along the line of the vertebral furrows the muscular cords project on the concave side; the only appearance of tension which is observed either on the concave or on the convex side of the curvature, arises from the merely occasional and temporary contractions of the muscles. When the deformity resists all attempts at rectification *(redressment)*—a case in which we might quite expect that the muscular cords should be still more prominent if the resistance was really owing to them—we usually perceive only a general tension of both sides of the spine, which becomes greater or less according as the muscles are in a state of contraction or of relaxation.

2. The curvature of the spine in lateral deviations is the result not of a real movement of the vertebræ on their axes, but of a peculiar *deformation,* or irregularity of formation of these bones, which takes place from the commencement of their pathological curvature. Until this *deformation* has occurred, there is no deviation of the spine, strictly speaking ; but only a temporary flexure which may pass away of itself, and which becomes a permanent curvature only when the osseous morbid change has taken place.

3. Curvatures of the spine continue after death, and even when the muscles of the back have been removed : the numerous skeletons of deformed persons in different museums abundantly prove the truth of this remark.

M. *Bouvier* concludes from his enquiries that the majority of lateral deviations of the spine are not produced by any muscular contraction, such as we observe to be the case in wry-neck, club-foot, and permanent flexures of any of the joints ; and therefore that the sub-cutaneous section of the muscles or tendons—so effectual a remedy against these deformities—is not applicable to the treatment of such deviations.—*Bulletin de l'Academie.*

*Remarks.*—When we first noticed M. *Guerin's* proposal in a recent number of this Review, we expressed our surprise that any educated surgeon could have dreamed of such an expedient for the relief of lateral curvature of the spine. It seemed to us to be the mere offspring of a most foolish love of notoriety, and that its adoption could never have been seriously contemplated by any one. It would seem, however, that M. *Guerin* has repeatedly performed the operation of dividing some of the muscles of the back, and, he says, with marked success. We should be very unwilling to permit him to experiment upon any of our patients.—*Rev.*

---

### On the relative Frequency of Herniæ at different Ages, &c.

M. *Malgaigne* has communicated to the Royal Academy the following details of his enquiries upon this subject. They are drawn from three series of observations, collected at the central Bureau of hospitals at Paris during the close of 1835, and during the two succeeding years.

The first series embraces 410 cases which were noticed in persons who applied at the central Bureau for trusses. Of this number, 335 were males and 75 were females.

The second series exhibits a total of 2,767 cases ;—of which 2,203 occurred in males, and 564 in females.

The third series comprises 2,373 cases ;—1,884 of which occurred in males, and 489 in females.

From these data we may reasonably conclude that ruptures are four times as frequent in the one *sex* as in the other.

Now with respect to the influence of *age* as a predisposing cause of hernia.

Among infants under twelve months of age herniæ are more frequent than during the second year of life. During the following three years the tendency becomes very strikingly less and less.

In considering the period of life between five and twelve years of age, it would seem that hernia is of more frequent occurrence during the latter than during the former half of this time. Hence we may infer that there is a *recrudescence* at about the ninth year or so. This *recrudescence,* or increase of predisposition, is still more marked during the next eight years—between thirteen and twenty. It is however to be particularly noticed that the increase bears exclusively on the male sex.

From the 20th to the 28th year, the number of herniary patients very sensibly increases, whether we take them in the mass, or distinguish them according to their sex.

Among men there is an increase of at least one fourth; and among women the increase is nearly one half. The rapid increase among males from the 13th to the 20th year of life may be attributed, in part at least, to the laborious occupations in which they are engaged. The same cause operates no doubt during the next eight years on both sexes; to which is now to be added the influence of marriage, and also of pregnancy.

From the 28th to the 29th year of life there is a notable augmentation in the disposition to herniæ—stronger indeed among women than among men; and indicative of some secret influence acting on the constitution, which becomes more decided in after-life.

The decennial epoch from 30 to 40 years of age may be divided into two halves, during the first of which the tendency to ruptures seems to remain nearly stationary, and during the second becomes considerably and suddenly greater—the average number for the series of the year 1836, being 29 in the first five years and 58 in the next five; and for the series of the year 1837, 26 for the first five years and 46 for the next five; shewing thus an increase of nearly a double.

During the next decenniad, from the 40th to the 50th year, the average decreases;—viz, from 58 to 54 for the series of cases for 1836, and from 46 to 42 for that of the following year. But here there is a marked difference in the relative frequency of herniæ according to the sexes. In the earlier years of life, the proportion of females affected with rupture was, as we have stated above, not more than a fourth of the number of males; but about the age of forty, this proportion rises considerably, some new cause of predisposition seeming to be operative about this period of life.

If, for example, we take the two preceding periods—from 30 to 35 years, and from 35 to 40 years—the proportion was found to be thus:

54 females and 231 males,
100 females and 418 males,

—the proportion being in either case short of a *fourth*. But during the next ten years, from 40 to 50 years, the number of women affected with herniæ was 242, while that of men was 722—giving a proportion of one *third*.

From 50 to 60 years of age, the general number of herniary patients increases, and the annual average becomes again equal to, or even above, what it was from the 35th to the 40th year. Moreover, the relation between the two sexes re-appears very nearly as during this latter period of life. From this we may infer that there is an increase of herniæ among men, and a decrease among women, during the period between the 40th and 50th year.*

During the seventh decenniad—from the 60th to the 70th year—the general number decreases; and as that of women seems to remain about the same, the proportion re-becomes nearly one *third*.

During the next decenniad, the number of herniæ in men was found to have decreased by not quite a half, while in women it had fallen by at least two-thirds. This difference seems to indicate, that at this epoch of life the mortality, which is evidently higher in herniary than in other persons, would be still greater from this cause in women than in men. If we follow the decrease of the herniary population, year after year, we no longer observe at this period—the eighth decenniad—as we had observed in the preceding ones, new cases of the disease to fill up the blanks caused by death. Thus, in the total of 3,140

* There is surely a mistake here; as we have been told in the preceding paragraph that there is a very marked increase of the complaint among women during the fifth decenniad of life. The decrease takes place in the next decenniad—viz. from the 50th to the 60th year, and not from the 40th to the 50th year.—(*Rev.*)

cases comprised in the two series, we find 48 in individuals of seventy years of age, only 22 of seventy-five years, six of eighty, five of eighty-one, three of eighty-two, and one of eighty-three years.

M. *Malgaigne* has lastly endeavoured to ascertain the proportional number of herniary persons to the entire population of France. According to his enquiries, there are about two in every 41 of the inhabitants. He has also tried to determine the relative frequency of the complaint in the different provinces: but his data on this topic are still very imperfect.

---

### On complete Absence of the Vagina.

The following case of this malformation recently occurred in the practice of M. *Manoury* of Chartres.

The patient was a girl, 17 years of age, who had never menstruated, but in whom, for a considerable time past, symptoms indicative of the menstrual secretion had been observed, returning at an interval of a month or so. Her general health had become much deteriorated, and various remedies had been fruitlessly tried, before the actual condition of the patient was ascertained.

On examining the external organs of generation, it was discovered that not only was there no external opening of the vagina, but that there was no discoverable trace of this canal existing between the bladder and the rectum.

A trocar was introduced for a short depth in the usual direction of the vagina, when a fibrous cul-de-sac was reached: this was traversed, and subsequently dilated by means of elastic-gum sounds. It is next stated, that 'the menstrual secretion *put prendre* its natural exit,'—(but whether it was really ever discharged is not mentioned. *Rev.*)—and that the cervix uteri could be felt with the finger.

Symptoms, however, of severe constitutional disturbance soon supervened; and the patient at length sunk under the effects of a constantly-recurring feverishness.

Unfortunately an examination of the body was not permitted.

M. *Capuron,* one of the most distinguished obstetrical writers in France, who reported to the Academy the preceding case, expressed his opinion that, where it is ascertained that the vagina is actually wanting—not merely imperforate—it is imprudent to have recourse to any operation, under the risk of compromising the life of the patient.

M. *Moreau* stated, that he was not prepared to assent to the opinion expressed by M. *Capuron,* viz. that no surgical operation should ever be attempted in such a case as the one under consideration.

It is quite true that *De Haen, Dupuytren, Dubois,* and others have failed, and that even fatal consequences have ensued from the bladder having been punctured in the attempt; but then, on the other hand, it is to be remembered that other surgeons, as M. *Amussat,* and M. *Wuillaume,* have succeeded. It is also to be considered that the deformity, if unrelieved, must inevitably prove fatal. If, therefore, the operation should succeed but once in a hundred times, we should not entirely abandon it.

M. *Velpeau* remarked, that the question under discussion is necessarily a complex one, as the lesions or irregularities of the vagina, which may occasion the complete occlusion of its passage, and the retention therefore of the menstrual secretion, may be referred to three different classes:—1. Where there is a congenital and total absence of the canal;—2. Where this occlusion occurs only at puberty or afterwards, in consequence of inflammation, &c.—3. The occlusion may exist in pregnant women, and may be the cause of serious impediment to the delivery of the child.

All will readily agree that, in young girls ander the age of puberty and in women advanced in life, no operation should ever be thought of; but, on the other hand, it is equally obvious that the case is very different when pregnancy exists, or when the catamenia are retained. Under the first of these conditions, no one would hesitate about dividing the obstacle; and many surgeons would assuredly at once adopt the same practice, when the catamenia only were retained.

M. *Capuron* admitted, as well as M. *Velpeau*, that an operation should be performed in those cases where there is a simple imperforation of the vagina; but that, if this canal be not only imperforate but wanting, and its place be occupied with a cellulo-fibrous cylinder, the propriety of an operation is more than doubtful.

Whatever be the opinion of surgeons on this subject, it should ever be kept in mind that the risk of the operation, where the vagina is really wanting, is very great; and therefore that all the circumstances of each individual case should be well considered before it is resorted to.—*Bulletin de l'Academie.*

---

### ON THE AMPUTATION OF THE PENIS.

Dr. *Barthelemy*, surgeon of the Hospital Gros Caillou at Paris, published in 1829 a memoir in which he recommends that an elastic gum catheter should be introduced along the urethra, and made to project well against the posterior parietes of the bladder, before the ablation of the penis is performed.

M. *Velpeau* and others had at first objected to this proposal that there was a risk of the end of the cut bougie slipping into the bladder. So far however from this being the case, it will rather tend to project or spring from the divided canal, provided its end has been pressed well against the posterior wall of the bladder.

It has been said by some writers, that there is no occasion to introduce the instrument until the diseased portion of the penis has been amputated, and that a catheter may always be passed into the bladder afterwards.

This is certainly not quite true. M. *Beclard*, in one case where he amputated the penis, could not succeed in introducing the catheter, and he was actually obliged to puncture the bladder. In another case, under the care of M. *Gimelle*, the patient died from infiltration of the urine. Whoever has seen many cases of this operation will acknowledge that he has seen great difficulty and much time lost in finding the divided orifice of the urethra, and in introducing a catheter along it, after the penis has been removed.

M. *Barthelemy* particularly calls the attention of the surgeon to the advantage of using a catheter that is sufficiently pliant, and of making it arch or project well against the posterior parietes of the bladder.

The assistant, standing on the opposite side of the patient, should press with his left hand the parts well against the instrument, keeping his fore-finger above and his thumb below the penis; while with his right one he holds firm its projecting end. The surgeon, standing on the left side of the patient, then amputates the member with one circular stroke of a small amputating knife.* The vessels may then be secured by *torsion;* and when all is done, the extremity of the catheter is to be securely fixed to the other parts of the bandages. One very great advantage of this mode of procedure is the celerity with which the operation—one which affects most patients more perhaps than any other—is finished.

Several successful cases are narrated; but it is unnecessary to particularise these.—*Gazette Medicale.*

---

* It is not expressly stated by M. *Barthelemy* whether he divides the catheter at the same time; but, from a remark in the report of one of the appended cases, we infer that he does.

### Epidemic Aphthous Inflammation of the Mouth.

This epidemic prevailed very extensively in the Foundling Hospital of Brescia in Italy during the Autumn of 1837. Its development was preceded by an epidemic of purulent ophthalmia, which had scarcely subsided, before the aphthous epidemic made its appearance among the children.

Its invasion was often so rapid and unperceived, that the whole surface of the tongue and inside of the mouth were found covered with vesicles in children, who had seemed quite well only a few hours previously. The precursory sign of this eruption seemed to consist in a more or less highly reddened state of the mucous membrane. The children became restless, refused their food, and began to pine. Often, while sucking, they quitted the nipple; and then the nurse usually experienced a sensation of burning in and around it. The colour of the tongue and gums became of a darker red, or of a livid hue. Then a few white points were observed on the apex and edges of the tongue and on different parts of the lining membrane of the mouth. These white points became gradually larger, and assumed the appearance of very minute mushrooms (*champignons*), resting on a narrow solution of continuity—hence this form of the disease has acquired in France the name of *muguet* or *millet.* When the white points extend in size without becoming at all elevated, and ooze out a whitish secretion, the disease has a more strictly aphthous character.

The greater number of authors have confounded these two forms of the disease together; but, according to Dr. *Girelli,* the reporter of the present remarks, they are different both in their origin and in the mode of their development.

The pseudo-membranous secretion was in some cases so widely diffused that not only was the entire surface of the tongue and of the inside of the mouth covered with it, but it even extended down the œsophagus. Here and there the mucous membrane of the cheeks exhibited a deep red, almost black, tint, and at the same time a remarkable dryness. There was also great heat, as might be readily discovered by introducing the finger into the mouth.

The lips and the tongue became hard, dry, and parched; the infant could no longer suck; and if it did but for a moment, the nipple of the nurse became hot and painful. In the bad cases, the extremities first and then the body became cold; and, before the death, the head sometimes felt as if the finger was placed on a piece of ice. Vomiting was not an unfrequent symptom; perhaps the inflammation had extended along the œsophagus to the stomach.

The duration of the disease was very various; from two to twelve days. The mere length of the disease seemed however to have little to do with its fatality, as many of the worst cases ran their course in two or three days.

From the preceding description, we may very properly designate this epidemic malady as one of *stomatitis aphthosa,* affecting more especially the follicles of the mucous membrane of the mouth.

The number of children under Dr. *Girelli's* care was 31; of which 14 occurred in September, 8 proving fatal; 7 in October, one only proving fatal; and 10 in October, all which recovered. It is a general character of such diseases that they are more severe at the commencement than towards the decline of the epidemic.

Dr. *Girelli* had reason to believe that the disease was readily communicated to healthy infants, by applying them to the same breast at which infants, already affected with the disease, had sucked. Hence the importance of separating the healthy from the sick, and of not permitting the same nurse to suckle both.

In the *treatment* of this disease, it is the first duty to ascertain whether the aphthous eruption in the mouth is associated with an inflammatory affection of any of the internal viscera; and, if such be the case, whether the eruption be primary and idiopathic, or secondary and consecutive.

Dr. *Girelli* strongly recommends the local application to the mouth of some emollient—as almond oil, or the syrup of succory with rhubarb. Recourse was also had to an emulsion of melon seeds, to which was added tartrate of antimony in minute doses. In this manner, the tendency to gastric disturbance was obviated, and a derivation, so to speak, was directed towards the intestinal canal. If it was necessary to act directly on the mucous inflammation, an emulsion prepared from quince-seeds with cherry-laurel water fulfilled this indication very well.

Smearing the aphthous surface with rose-honey, or with a solution of borax in marsh-mallow water, was often useful. But perhaps one of the most useful and necessary of all remedies was the application of one or two leeches about the angle of the jaw. Dr. *Girelli* assures us that he has derived much more benefit from cautious local bleeding than from almost any other means. In some cases, the application of a blister to the nape of the neck was of service.

It is of the highest consequence that the child, if at the breast, should be suckled by a healthy sound nurse, and, if not at the breast, that it should be fed with light nourishing food. One nurse should never suckle more than a single child. To shew the importance of this precept, we need only mention that, in the Foundling Hospital at Brescia, the number of patients and the amount of the mortality were always proportionate to the fewness of the nurses.—*Annali Universali di Medicina.*

---

### ON THE INFLAMMATIONS OF THE CÆCUM.

The high character of the author of the following remarks, Frederick Albers Professor of Medicine in the University of Bonn, is a guarantee for the exactitude of the observations and the soundness of the reasoning.

That the diagnosis of diseases of the cæcum is attended with no inconsiderable difficulty appears from the well known circumstance, that very often they have never been suspected to exist during the life of the patient, and have been discovered only on dissection.

In the following observations we propose to treat *first,* of acute inflammation of the cæcum; *secondly,* of inflammation of the cellular membrane which surrounds it; *thirdly,* of the inflammation of the cæcum produced by the accumulation of fæcal matters, or the presence of some foreign body in the gut; and *lastly,* of chronic inflammation of the gut.

1. *Acute Inflammation of the Cæcum.*—Most frequently it commences in the mucous membrane during the progress of dysentery and enteritis; but sometimes however it arises idiopathically, and seems to be quite independent of any other complaint.

In the latter set of cases, the inflammation is usually very rapid and intense, accompanied with the following symptoms;—a burning pungent pain in the right iliac fossa, aggravated by pressure and by intestinal evacuations, and frequently extending more or less along the line of the transverse colon. A sensation of burning heat at the anus every time that the bowels act is a common attendant symptom.

There is usually a distressing diarrhœa present: while this lasts, the pain in the iliac fossa is generally diminished. The stools are thin, often loaded with mucus, and not unfrequently bloody. As the disease abates, the mucosities become whiter and of a thicker consistence, not unlike to what are often expectorated in bronchitis. This mucous excretion has frequently been mistaken for, and described as, purulent; and hence a common error, that in this affection the stools are often mixed with matter. Every pathologist however knows well how very rare it is for inflammation of any portion of the intestinal canal to terminate by suppuration.

With respect to the pain which accompanies inflammation of the cæcum, it deserves notice that this usually extends down in the direction of the right limb, more especially when the patient walks or turns his body round in bed. Hence it is apt to be considered as of rheumatic origin ; and, as the muscles covering the inflamed bowel generally sympathise with it, there may indeed be a certain degree of rheumatic suffering blended with that arising from the enteritic disease. In some cases retraction of the right testicle has been noticed; and in others symptoms of irritated kidney occur.

*Duration.*—Professor *Albers* has never known a case of acute inflammation of the cæcum to last longer than seven days.

*Inflammation of the Cellular Substance around the Cæcum* usually commences very suddenly after exposure to cold, irregularity of diet, or taking a draught of cold liquid when the body is heated and perspiring. The pain, which attends it, is felt at first sometimes near the umbilicus, and at other times in the iliac region. When it begins around the navel, the patient usually complains of slight cutting pains, which do not differ much from ordinary colic: when in the iliac region, it is much more intense. Wherever it is situated, it gradually diffuses itself, so that soon the entire surface of the abdomen becomes exceedingly tender, as is the case in genuine peritonitis. Sometimes the pain extends round to the loins and back, and then the case may be mistaken for nephritis or psoitis. But at length it is concentrated, chiefly in the iliac fossa. When this takes place, the disease is *franchement declarée*, and probably suppuration is nearly at hand. The pain is aggravated by any movement of the body, or by the accumulation of flatulence in the bowels, &c. &c.

When the pain has lasted for some time there is always considerable tension, swelling and hardness in the right iliac region, stretching from thence in all directions, but chiefly downwards in the direction to Poupart's ligament. These phenomena are more circumscribed than in peritonitic inflammation. But it must be admitted that the two cases are sometimes not easily distinguishable. Read the following, for example :—

A child, eight years of age, was suddenly seized, after a chill, with smart fever attended with severe abdominal pain, which was seated at first in the epigastrium, and gradually extended itself to the right iliac region, and finally over the whole abdomen.

There had been diarrhœa ; but this was replaced by constipation and troublesome vomiting. The case was considered as one of genuine peritonitis, and treated with bloodlettings, local and general, fomentations, mercury internally and externally, &c.

The patient died on the ninth day after the attack.

*Dissection.*—The whole extent of the peritoneum, intestinal as well as abdominal, was perfectly sound, with the exception of one spot of about the size of a crown-piece over the cæcum, where it was evidently inflamed and exhibited a few flocculi of coagulable lymph. On examining more minutely the parts at this region, a fluctuation was perceptible; and on making an incision there, a large cupful of purulent matter flowed out. The cellular tissue, surrounding the cæcum behind, was found greatly destroyed by suppuration, and the pus had made its way between the abdominal muscles forwards to the iliac region. These muscles were quite dissected, as it were, from the subjacent peritoneum; the cæcum also was much softened in texture, so that it was easily torn across; and its mucous surface was of an almost livid colour: no perforation, however, had taken place. All the other abdominal viscera were sound.

It will be observed that, in the case now related, there was diarrhœa at first, and that this was followed by obstinate constipation. Now such is the usual occurrence, whenever the cellular substance around the cæcum becomes inflamed.

The constipation, which often resists every remedial means that are employed, is probably owing partly to the muscular coat of the gut, which is in close proximity with the inflamed cellular tissue, becoming affected itself,—and *Abercrombie* has shewn that inflammation of the muscular tissue of a gut paralyses its functions—and partly to the mechanical effect of the swelling not only on the cæcum, but also on the colon and small bowels.

These two causes render this conspitation much more obstinate than in almost any of the ordinary forms of enteritis.

Besides this and the other symptoms already enumerated, there is usually pain and a sense of numbness down the right thigh and about the hip-joint. These symptoms may be owing to the psoas and iliacus internus muscles being peculiarly affected. The excretion of the urine is also in many cases more or less disturbed. It is probable that the right kidney sympathises from the very commencement of the malady, and that the swelling in its latter stage may press upon the ureter.

We need scarcely add, that there is always more or less feverish irritation of the constitution present. The progress of the case is indeed often very obscure and slow; the symptoms being at first inconsiderable, but often becoming on a sudden violent and most alarming. The *duration* of the disease may be said to vary from two or three weeks to several months, or even to upwards of a year.

*Termination.*—It is not rare to find that inflammation of the cellular substance around the cæcum terminates favourably by resolution; but certainly in the majority of cases suppuration is induced.

The pus usually finds its way into the cavity of the gut, either directly or by bursting into the appendix vermiformis. When this takes place, there is a sudden change in all the symptoms; the severe pain and the obstinately confined state of the bowels being generally followed by a complete remission of suffering, and by a greater or less degree of diarrhœa; so that the patient, and his medical attendant also if he be not on his guard, are apt to suppose that a favourable crisis has taken place. Too often, however, this is but a delusive calm; the strength of the patient becomes weaker and weaker; the stools are found to be mixed with purulent matter, and the system at length gives way.

The history of the following case affords a good illustration of the usual progress of the disease.

*Case.*—A man, 29 years of age, who had been previously in perfect health, was seized, during the Summer of 1833 when the influenza was prevailing, with smart abdominal pains, which returned at periodic intervals, but were not attended with fever or any gastric disturbance; diarrhœa however was present. By the use of cupping the pains abated, but they became more permanent and more fixed in the right iliac region; and at the same time a constipated state of the bowels ensued. For five days the patient was able to attend to his affairs; and then he was seized with feverish chills followed by flushes of heat. The pain became much more severe, and occasional vomitings supervened; at the same time the groin was somewhat swollen and very tender on pressure, and the urine was thick and very red. Purulent matter was observed to be mixed with the alvine dejections; all the symptoms became suddenly very alarming, the extremities being cold and the pulse scarcely perceptible; and the patient died in a state of coma.

On dissection, a large collection of pus was found behind the cæcum, stretching up to the right kidney, and down to the pelvis; the appendix vermiformis was hard and thickened. In the cæcum, at about an inch from the appendix, there was a perforation with irregular edges, through which the matter had escaped into its cavity; the right kidney was softened and very red; and the iliacus internus muscle was partially destroyed by ulcerative absorption.

In a few rare cases, the pus makes its way not only into the gut, but also outwardly through the abdominal parietes; thus an anus *contra naturam* is established. Occasionally the outward opening alone takes place. When this is the case, the symptoms are generally very severe for ten or twelve days, and then suddenly they subside, when the abscess bursts. Under all circumstances the disease must always be considered as a very dangerous one; the colliquative exhaustion that is induced by the protracted suppuration, proving in most cases fatal.

With respect to the age, at which *peri-cæcal* suppurations are most frequent, perhaps it is that of youth.

### *Stercoral Inflammation of the Cæcum.*

The ingenious researches of Dr. *Schulze* of Berlin have shewn that the cæcum exercises an important part in the act of digestion, and that it is not without reason that this portion of the large intestines has been designated the *ventriculus secundus.* In some individuals its sacciform shape and arrangement must increase the tendency to an accumulation of fæcal matters within its cavity; more especially when there is at the same time an excess of its chief curvature, or when any irregular adhesions exist to retard the onward movement of its contents, or lastly when the ascending portion of the colon is more than usually arched or bent. Persons who have much sedentary occupation, who live chiefly on a vegetable diet, and in whom there is a tendency to constipation, are those who are most subject to this complaint.

The fæces gradually accumulate in the cells of the cæcum, until a process at first of irritation, and subsequently of inflammation, is excited, and then all the symptoms of enteritis or of strangulated hernia are excited. Dr. *Posthuma* of Groningen has expressed his opinion that atony of the gut is the primary cause of the mischief, (*de cæci, ejusque processus vermicularis pathologiâ, Groningen* 1836), and that in consequence of this it loses the power of propelling its contents onwards, which thus accumulate more and more until at length the coats of the bowels become inflamed. In such a case, an obstinate constipation is perhaps the only symptom that is present for a great length of time, and the physician may have no grounds to suspect any local mischief until swelling or pain is felt in the right groin. The subsequent progress of the case may be understood from what we stated in the preceding section. We shall therefore give only a brief extract from the author's remarks on the appearance of the abdomen in this disease.

"The abdomen is usually tense, hard, and prominent, especially in the right flank. If we attentively examine this part, we shall generally find that it is the seat of a large swelling, which can be displaced more easily from one side to the other from above downwards. At first, light frictions over this part will probably cause the swelling to disperse; not so, when the malady is farther advanced. Pressure on the part usually causes a certain degree of uneasiness, but rarely any acute pain. In the left iliac region, the descending colon may often be felt filled with indurated fæces. A sense of pain and numbness is not unfrequently felt along the line of the *iliacus internus* muscle from the groin downwards along the inner side of the thigh; this feeling usually becomes more and more distressing as the complaint advances." He adds—

"A dull sound is elicited by percussion of the right iliac region."

The various symptoms, which attend this disorder, may exist for several days before they come to any crisis. Professor Albers has observed in some cases that the most conspicuous symptom for a great length of time is a most troublesome itching of the surface. If a diarrhœa should take place, whether spontaneously or from the use of purgative medicines, the symptoms are usually rapidly and effectually relieved.

It is truly astonishing what enormous quantities of fæces have been discharged before entire relief is obtained.

There is no doubt but that this complaint—irritation of the cæcum from accumulation of fæces—may in most cases terminate favorably. It is only in a few rare instances that from some cause or another there is a permanent lodgment of the fæces in the cavity of the gut—either from paralysis of its muscular tunic, or from some obstruction in the colon or rectum—and that this accumulation excites an inflammatory and perhaps at length a suppurative action in its walls or in the adjacent cellular tissue. When the abscess bursts outwardly, fæculent matter is not however in all cases mixed with the purulent discharge; as the gut may perhaps never have been perforated, or the aperture may have been so small that it has subsequently closed up.

Such cases not unfrequently terminate well, the suppuration gradually diminishing and the abscess at length healing up. In a few rare instances, the inflammation has terminated in gangrene. The following one occurred in Professor Albers' practice.

An old man, who had been for a length of time affected with Prurigo senilis and with a most troublesome constipation, was suddenly seized, after a full meal, with the symptoms of an enteric *phlegmymenite.* (What does this new word mean? we have never met with it before.—*Rev.*) The pain was chiefly in the right groin, which was tumefied; the patient had frequent calls to go to stool, but he could not satisfy them. For four days there was no relief of the bowels. Vomitings came on. On the seventh day, the patient became delirious, then comatose, and died.

*Dissection.*—The cæcum was found so much altered in its tissue that it gave way on the slightest pressure. On its internal wall there was a perforation, through which some fæcal matter had escaped into the abdominal cavity; the cæcum itself contained a quantity of fæces, and not fewer than 27 kernels of plums. All the adjacent parts presented traces of severe inflammation.

Finally, we may state that, in a few instances, the life of the patient has been much prolonged by the establishment of an *anus contra naturam* in the right groin.

As affording an illustration of many of the foregoing remarks, we shall briefly report one case more.

A man, 72 years of age, died last year in the Salpetrière at Paris, after protracted suffering from intestinal disturbances. The inferior part of the sigmoid flexure was much contracted; and the consequence of this lesion had been that an immense quantity of fæces, in large hard lumps, was accumulated in the transverse portion of the colon, which might actually be felt through the thin parietes of the abdomen; the liquid parts seemed to have been, as it were, filtered through them, and hence during life, the patient had supposed that he had a diarrhœa. He died suddenly and without experiencing much pain. On dissection, a large ulceration was found in the cæcum, and a cancerous contraction of the sigmoid flexure.

In another case—which terminated favourably—where the rectum was obstructed by an encephaloid tumor, the fæces had accumulated in large hard balls in the sigmoid flexure and descending portion of the colon. The cæcum and rest of the colon were distended with gas. This state had continued for upwards of a twelvemonth, when suddenly the abdominal parietes became the seat of sharp pains, which were most severe in the right groin. By the use of purgative medicines, an immense quantity of the large fæcal balls was discharged, and the patient was speedily relieved.

In conclusion, we may direct the reader's attention to an essay by Dr. *Ferral*, in the Edinburgh Medical and Surgical Journal for July, 1831.—*Beobachtungen auf dem Gebiete der Pathologie.*

---

### Cases of Operation for Artificial Anus in the Left Loin; Discussion at the Royal Academy.

*Case.* 1.—A middle-aged woman, who for many years had been subject to constipation, but who had long enjoyed perfect health with the exception of some difficulty in defecation, was at length seized with so obstinate an obstruction of the bowels that all means failed to procure an evacuation. For 26 days there was no relief, and the patient suffered from the most excruciating torments in the abdomen. M. *Barras*, then M. *Amussat*, and lastly a consultation of these two gentlemen associated with MM. *Fouquier*, *Recamier*, *Breschet* and *Puyoo*, were called in to the case.

M. *Amussat*, having pushed his finger up the rectum as high as it could possibly go, felt a round and firm tumor, which seemed quite to plug up the gut. This result, says the reporter of the case, was particularly confirmed by M. *Recamier*, "whose great precision of touch so often enables him to form the most unexpected diagnosis." As all medical means had failed, it was resolved to give the patient a chance of recovery by forming an artificial anus in the left lumbar region. MM. *Recamier* and *Breschet* at first wished that the bowel be opened in the left groin; but, M. *Amussat* having convinced them of the practicability of performing the operation in the left lumbar region, they assented to his proposal.

The following is the description of the operation which M. *Amussat* performed: "A transverse incision, four inches and a half long, was made in the left loin, extending from the edge of the sacro-lumbaris muscle to about two fingers' breadth from the upper crest of the os ilii. The trajet of this incision comprised the entire thickness of the abdominal muscles and the adipose-cellular tissue, in which the kidney is enveloped, and by which the posterior surface of the lumbar colon is attached. This intestine being extremely distended with fæces—so much so indeed, that there was quite a prominent fulness on this side of the loins before the operation—protruded between the lips of the wound. It was readily recognised by the arrangement of its fibres and by the purplish colour it exhibited. Two arteries were secured by torsion, and a needle, armed with a waxed ligature, was passed through the most prominent part of the gut in order to prevent its receding upon being opened. A trochar was then plunged into it; a quantity of gas and of liquid fæculant matter escaped.

The opening was enlarged by a probed bistoury to the extent of an inch and a half. Immediately, the fæces were discharged in great abundance and in an almost continued stream, until three vessels were filled. Injections of tepid water were passed both up and down the gut, by the use of which a considerable quantity of tolerably consistent fæces was washed out. The edges of the gut were then secured to the lips of the wound by five stitches, and a large poultice was laid over all."

It is unnecessary to follow the report in its subsequent details. Suffice it to say that the patient gradually recovered, the artificial anus continuing to perform its duty about twice a day. Nothing but the gaseous contents of the bowels escaped by the natural anus. Four months subsequently, the patient continued well.

*Case* 2.—A man, 62 years of age, had for a length of time been subject to a most constipated state of bowels and to piles. The act of defecation was almost always attended with difficulty and pain; and occasionally the rectum was so much obstructed by the accumulation of hardened fæces that it was requisite to extract them. Usually blood was discharged along with each stool; and, for two or three years past, the dejections were frequently streaked with mucosities and purulent matter.

Dr. *Foville,* who was first consulted, ascertained that about an inch and a half up the rectum there was a carcinomatous tumor, which blocked up the gut so much that the fore-finger could scarcely be made to pass through. A consultation consisting of MM. *Recamier, Breschet* and *Amussat* was called. It was resolved to attempt the destruction of the tumor by crushing with long forceps the projecting granulations of the cancerous ring (*bourrelet*)*. This was done by M. *Amussat.* When the blood and bruised portions of the tumor were removed, a stream of cold water was injected up the gut with the view of preventing the accession of inflammatory mischief.

Eight days after the *broiement,* it was determined that recourse should be had to cauterization with potassa, with the aid of the speculum. This was repeated seven times, at intervals of three or four days; the cold lavements being repeated after each application. The effect of the treatment now described was to reduce the tumor to one half its original bulk.

The condition, however, of the patient became gradually worse. For ten or twelve days at a time the bowels would not act, and when an evacuation—or, as the French term it, a *debacle*—occurred, the patient's strength was quite exhausted. The symptoms became so alarming that his speedy death seemed inevitable, unless some surgical operation was performed to provide a mode of relief for the bowels.

It was resolved, in a second consultation, that an artificial anus should be established as in the preceding case. It was performed as follows, by M. *Amussat.*

"A transverse incision, four inches and a half in length, was made at about a finger's breadth from the spinous processes of the vertebræ, and midway between the last rib and the crest of the os ilii. At the anterior angle of the incision was observed the sac of the peritoneum, beneath which the small intestines were visible. The lumbar colon was found to be strongly contracted upon itself, and to be in a great measure covered by the *quadratus lumbi* muscle, which it was therefore found necessary to cut across. The intestine, being seized, was opened with a bistoury; nothing but gas and a few fæcal bullets escaped.

The edges of the gut were secured to the lips of the wound with four stitches; the latter being subsequently brought together at each angle by three points of the twisted suture. For five days subsequently, scarcely any thing escaped from the artificial opening; and the injections of water which were thrown into the colon returned altogether by the natural anus.

The artificial anus was, however, gradually enlarged by means of prepared sponge, elastic-gum and wax bougies. The effect of this was that the escape of fæcal matters was greatly facilitated; and, as this discharge took place, the general health of the patient was much improved. The stools became gradually regularised, and the fæcal bolus was at length expelled from the artificial opening in a cylindrical form."

The tumor in the rectum remained stationary, with the exception of being of harder consistence.

---

* We must reprobate such practice as positively murderous, if the disease be genuine cancer. Our readers may remember some remarks of ours on this fatal error of French surgery in our notice of the last illness of the celebrated *Broussais* in the last number of this Review, M. *Amussat* was the surgeon in that case too.

*Discussion on the preceding Cases at the Royal Academy.*

M. *Velpeau,* while he admitted the great merit of his colleague M. *Amussat,* in having been the first to prove the safety of *Callisen*'s operation for artificial anus in the left loin or flank, was still inclined to give the preference to the older one recommended by *Littré,* even though the peritoneum is thereby necessarily opened—whereas in the other method this is avoided—and which consisted in opening the colon in the left iliac fossa. He himself had performed it once in an old woman, who however died two days afterwards. *M. Syme,* of Edinburgh, lately performed the same operation in a case of cancerous tumor of the rectum, and his patient survived for sixteen months; and in *Markland*'s case, life was prolonged for upwards of four months.

One important advantage of Littré's operation is that the artificial anus is situated in a more convenient position, the patient being able to attend to it himself; whereas this is almost impossible, if it be in the loin.

M. *Velpeau* gave credit to M. *Amussat* for the modification which he had introduced into *Callisen*'s operation—viz. the substitution of the transverse in place of a vertical incision in the loin.

M. *Gerdy,* however, seems to give the preference to the latter line of incision, as recommended by Callisen. He alluded to the extreme difficulty of the diagnosis in cases of diseased rectum, which may induce the necessity of an operation for artificial anus, and suggested that, whenever there is any uncertainty in the diagnosis, the sphincter ani should be freely divided to enable the surgeon to pass his finger up the gut as high as possible.

M. *Amussat* expressed himself opposed to the suggestion of M. *Gerdy.* In his opinion, the finger may in most cases be pushed up very high, especially if an assistant presses up the elbow of the surgeon at the same time.

M. *Breschet* spoke in terms of the highest approbation of the ability of his friend M. *Amussat* in planning and in performing the operation in the two cases which have been described. He had assisted at both. He frankly admitted that he had been inclined to *Littre's* operation, until he had witnessed, first, the experiments by M. *Amussat* on the dead body, and, subsequently, the two operations on the living. He had himself made numerous examinations of the relative anatomy of the colon in the left loin, with the view of determining whether at this part there was usually a mesocolon or not. The result of his enquiries was, that in some cases the peritoneum invested every part of the intestines; and that, in other cases, a small portion of its posterior surface was left uncovered.

The extent of the uncovered surface, however, depended greatly on the circumstance whether the gut was distended or empty. But even when a distinct mesocolon was present, he was of opinion that its layers might be easily separated either by the handle of the scalpel, or by blowing into the cellular tissue between, and that thus the intestine might be opened, without necessarily wounding the peritoneum. He decidedly preferred the transverse incision of the abdominal parietes practised by *Amussat,* to the vertical one as recommended by *Callisen.*

M. *Blandin* concurred entirely in the opinions of the former speaker. He thought that great merit was due to his colleague M. *Amussat,* for having by his boldness and talent rescued the operation of *Callisen* from oblivion, while at the same time he had decidedly improved it by one or two important modifications.—*Bulletin de l'Academie.*

### On Arsenic in the Bones of the Human Body.

M. *Orfila* recently submitted the results of some researches on the above subject to the Royal Academy.

The following is the method by which he has succeeded in detecting the presence of a minute portion of arsenic in human bones, in a natural state.

The bones are to be first calcined, but not by too high a heat, and they are to be carefully preserved from any contact with the fuel employed. The pulverised mass, being previously sifted, is to be treated with *purified* sulphuric acid; the mixture is then to be introduced into Marsh's apparatus. Spots or stains of arsenic, having a brown colour, and a metallic brilliancy, will soon be perceived on the sides of the vessel.

M. *Orfila* has succeeded in detecting minute portions of arsenic in the bones, not only of the human adult, but also of the sheep, ox, and horse. Hitherto he has never discovered any traces of its existence in any of the soft parts of the animal body.

He seems, however, to believe that there may probably be a very small portion in muscular substance, although hitherto he has quite failed in detecting its traces. Some of the appearances, however, observed during its slow distillation have been not unlike to those which are known to proceed from the presence of arsenic.

---

### On Incontinence of Urine in Children.

*Treatment.*—The late Baron *Dupuytren*, and also MM. *Baudelocque* and *Guersent* have recommended the use of cold shower-bathing as one of the most effectual remedies against this most annoying and frequently most obstinate complaint.

M. *Lallemand*, of Montpelier, has great confidence in aromatic bitters to which a small portion of brandy has been added, followed by active friction of the loins.

*Underwood* recommended the use of sea-bathing, of dry cupping, of blisters on the sacrum, and of electricity.

As internal medicines, the Spanish fly and the nux vomica have been unquestionably the most efficacious. The preparation of the latter, which has been most successfully used, is the extract in doses of from half a grain to four grains in the course of the day. The following two cases may be read with interest.

*Case* 1. A girl, 12 years of age, had been affected from her infancy with incontinence of urine, her general health being unaffected all the time. It would seem that no remedial means had ever been tried.

Dr. *Ramaugé*, who accidentally saw the girl at a house where he was visiting, recommended her to take one of the following pills, along with a wine glassful of infusion of Quassia, three times a day:—

℞. Extracti nucis vomicæ gr. viii.

Oxydi ferri nigri ʒi.

Pulv. Quassiæ ʒi.

Syrupi absinthii q. s.

In pilulas xlviij. divide.

A tonic nourishing diet was ordered, and also a glassful of wine two or three times a day. By persevering in this course for a month, the patient was quite relieved from her distressing malady.

The treatment was, however, continued for another month; and at the date of the report there had been no return of the complaint for upwards of a year.

*Case* 2. A boy, ten years of age, had long been affected with nocturnal incontinence of urine. During the day he had very frequent calls to urine, the bladder being unable to retain only a very small quantity at a time. He was ordered an infusion of Quassia and half a grain of extract of nux vomica in four pills during the course of the day. After three weeks' employment of this regimen, the boy could very sensibly retain his urine a great deal better, the calls being much less frequent. A blister was applied upon the sacrum, and a cold aromatic bath was to be employed twice a week. In the course of a fortnight, the nocturnal incontinence had quite ceased, and the patient continued well for six months.—*Journal des Connoissances Medicales.*

---

### On Acute Glanders.

From a very able report, by MM. *Nonat* and *Bouley*, on the recent work of Dr. *Deville* on Glanders, we shall extract a few passages.

After admitting that the transmission of the disease, from the horse to man, was recognised and admitted in Britain, Germany, and the United States, for several years before the French practitioners gave in their consent, they go on to state:

"Dr. *Rayer* had read with the greatest interest the very remarkable memoir of Dr. *Elliotson*, when, in February 1837, a groom was accidentally admitted into the Hôpital de la Charité, affected with typhoid symptoms, subcutaneous abscesses, and a pustular and gangrenous eruption on the skin.

This patient died; and, on dissection, numerous pustules were found dispersed along the nasal passages and in the larynx, and several small abscesses were discovered imbedded in different parts of the lungs. M. *Rayer* had at once predicted the nature of the disease during life: and his opinion was quite confirmed by the results of the necroscopic examination. He ascertained subsequently that this man had, for some time, been in the habit of sleeping in a stable, where several glandered horses were kept."

When the report of this case was communicated to the Royal Academy of Medicine, it met with the most animated opposition from the veterinary surgeons who have a seat there. M. *Rayer*, however, was nothing daunted; indeed, this very opposition induced him to prosecute his enquiries with redoubled zeal.

Aided by M. *Leblanc*, a very intelligent veterinary surgeon, he applied himself to study the disease of glanders, and other cognate morbid affections, as they occur in the horse; and the result of his enquiries was, that he came to the conclusions that—

"The glanderous principle shews itself in solipedous animals under four different forms—the acute glanders, the chronic glanders, the acute farcy, and the chronic farcy;—and that each form may exhibit a variety of modifications from the union, in one case, of the characters of several together."

Having established these principles, he then set himself to collect all the cases on record of the alleged transmission of glanders from the horse to man, and to arrange them under the four principal forms now mentioned.* These

* The chronic form of glanders, when it affects men, is infinitely less frequent than the acute, even when the disease in the horse, which gave the infection, was of the chronic nature.

are all detailed at length in the memoir, which he published, and which has excited so much attention among all practitioners, veterinary as well as medical. Within the space of a year, several new cases were observed and reported by some of the leading physicians and surgeons of the metropolis, as MM. *Breschet, Andral, Husson, Jobert, Roux,* and others. In all of them the symptoms, characteristic of the disease, were observed in men who had had to do with diseased horses.

MM. *Nonat* and *Bouley* have subsequently established the important fact, that the glanderous principle is communicable from man to the horse, as well as from the horse to man. M. *Nonat,* having met with a patient affected with acute glanders, inoculated a sound horse with some of the discharge from an abscess which had formed over the right parietal bone; and the result was that the horse became diseased.

As the case is a very interesting one, we shall give some particulars of it.

In February 1839, a young man, 21 years of age, was admitted into the Hôtel Dieu, under the care of M. *Nonat.* He was in a state of great prostration of strength, and highly feverish; his breathing was rapid and oppressed; the skin was hot, the face flushed, the eyelids swollen, and the eyes themselves glazed. The right side of the forehead was red, painful and puffy; further back there was a swelling on the scalp, which, when pressed upon, conveyed to the finger a sense of fluctuation. Other two abscesses existed; one on the right thigh, and the other on the left leg. Next day several pustules made their appearance on several parts of the body; and now, when the nostrils were compressed, a sanguineous frothy fluid exuded out. This pressure caused pain; and at the root of the nose the integuments began to exhibit a red and swollen appearance. During the night a purging came on, and the patient was excessively restless and uneasy.

On the following morning there was a drowsiness, which was speedily followed by a state of complete coma; different portions of the scalp and face had become of a livid aspect; other abscesses had made their appearance in various parts of the body, and there were fresh pustules on the arms, legs, and chest; a thick yellowish-white discharge also oozed from both nostrils. All these symptoms continued to increase until next day, when death took place.

*Dissection.*—The body was covered with pustules, which were depressed on the surface *(affaissées,)* without any redness at their base, and of a dull white aspect: some were ulcerated.

The alterations of the lymphatic system were in strict relation with the pustular eruption on the surface. The ganglia, which were the most diseased, were those in the armpits and groins.

In addition to the abscesses noticed above, there were found many others in various states of maturity; some having the appearance of diffused purulent collections, but the greater number being very similar to what are well known under the name of metastatic abscesses. The veins of the dura mater and the cells of the diploe presented many traces of marked inflammation.

The mucous membrane on the septum narium was swollen and highly injected with blood: here and there dark patches of ulceration, and several pustules also, were observed. On the floor of the nostrils the mucous membrane was found thickened, very much softened, and covered with a gray-coloured *detritus:* the maxillary sinus was full of a yellowish viscid mucus.

On the surface of the lungs there were numerous ecchymosed spots; and imbedded within their parenchyma were several abscesses—under the form of circumscribed pulmonary hæmorrhages, having a purulent infiltration in their centre. The largest of these were of the size of a walnut, the smallest of that of a hazel-nut.

*Remark.*—It was with the matter taken from one of the abscesses of this patient that M. *Bouley* inoculated two horses: it was inserted at the margin of the nasal passages.

In one of them the acute form of the disease very quickly shewed itself, and the animal died on the eighteenth day, after having exhibited all the usual symptoms of decided glanders.

The other horse lived for twenty-eight days, and then died very suddenly; the immediate cause of death being certainly a rupture of the aorta; although many of the symptoms of acute glanders had been manifested during life, and also some of the most pathognomonic traces of this disease also were discovered on dissection.

To add further weight to this conclusion—that the glanderous principle may be communicated from man to the horse, we may state that MM. *Nonat* and *Bouley* inserted some matter taken from one of the horses, which had been inoculated from their patient, into another horse, and that this last animal quickly sickened, and eventually died with all the symptoms of the genuine disease.—*Revue Medicale.*

---

### Memoranda on the Treatment of Apoplexy.

Dr. *Jourdain*, the author of the following observations, very justly remarks in reference to the treatment of all diseases, that "no sound therapeutics can exist without an accurate knowledge of their pathology, or, in other words, of their organic causes. Without such a knowledge, practical medicine is nothing better than a methodic empiricism." It must be acknowledged that even in the management of not a few maladies, whose real nature and cause are tolerably well known, it is impossible to lay down any definite line of practice, applicable to every case and to all circumstances. Of no malady is this remark more strictly true than of the hæmorrhagic apoplexy of aged people. The too common practice of at once resorting to blood-letting, whenever the supposed symptoms of this disease are present, is now admitted by almost every intelligent physician to be alike most unscientific in theory, and most pernicious in practice.

Before examining this point we shall very briefly mention the views, which seem to us the most rational, as to the nature of *encephalorrhagia* in old people.

Cerebral hæmorrhage in old people results from the rupture of a blood-vessel or blood-vessels, which have become altered in their texture, either from cretaceous concretions in their parietes, from ulcerations, or lastly from the softening of the cerebral substance itself. If apoplexy resulted from a simple sanguineous exhalation, as many physicians seem still to believe, it would surely be more frequent in young than in aged persons. But this we know to be certainly not the case: genuine *encephalorrhagia* being very rare in early and in middle age. Again, how often does an attack of apoplexy take place most unexpectedly, when the patient was, almost the very minute before, in seeming perfect health, and at a moment when his circulation was quite tranquil and undisturbed! The disease may in some instances be induced by causes, moral as well as physical, which tend to increase the rapidity and impetus of the cerebral circulation; but it is equally true that very often it suddenly occurs without any such premonitory phenomena. In a few instances indeed the blood-vessels of the brain have actually been found on dissection to be nearly empty of blood. (*Abercrombie.*)

With respect to the *causes* of cerebral congestion, Dr. *Jourdain* suggests that they may be well arranged in the following manner:—

| | | |
|---|---|---|
| A. Encephalopathy | Idiopathic | Mental affections.<br>Alterations of the Blood. |
| | Sympathetic | Indigestion.<br>Constipation.<br>Sexual intercourse, &c. |
| B. Obstacles in the course of the blood | | Diseases of the heart.<br>Influence of cold on the surface, &c. |

He then proceeds to offer some remarks on the treatment of the various forms of cerebral congestion, which arise from the different various causes now enumerated. He is, we may remark, decidedly opposed to the detraction of blood, when the disease is manifestly attributable to any intense mental or moral emotion of the mind, as fear, surprise, grief, or excessive joy, remarking: "It is either by the intestinal discharges or by cutaneous transpiration that nature seems to obviate the effects of fright. Tears, it is well known, are the natural relief of grief. Again, a sudden diarrhœa will often save from convulsions infants, when their nervous system is much excited by painful dentition. In short, the secretory and excretory systems seem designed to give an exit to the nervous fluid, when it has become suddenly accumulated in any of the internal organs. If, however, severe mental emotions should induce inflammatory phenomena, it may be necessary to have recourse to blood-letting to relieve the symptoms. But on the whole, it is better to employ in most cases soothing medicines, rubefacients, &c. and especially endeavour to imitate nature by promoting certain secretions. *Quo natura ducit, eo ducendum.*"*

Passing over Dr. *Jourdain's* remarks on the influence of alterations in the condition of the blood itself, and also of indigestion and constipation, in inducing cerebral congestion, we proceed to what he says of the treatment of those cases, where the disease is connected with organic disease of the heart—his object, be it remembered, being all along to point out the inefficiency and danger of blood-letting in many of the forms of apoplexy.

Alluding to the opinion that hypertrophy of the heart is a frequent cause of cerebral disease—an opinion, by the bye, which has been most perseveringly combated by M. *Rochoux*—he quotes passages from several distinguished writers who have pointed out the evils of indiscriminate blood-letting. The following extract from *Andral* is certainly pertinent to his purpose:

"The mere size," says this distinguished pathologist, "of a muscle is not the only condition or element of its energy. If this be true, the reader will perhaps at once perceive how much it must influence our medical practice in the treatment of diseases of the heart in aged people. Bloodlettings should not be employed in their case but with great caution, and only as a mechanical means to relieve the oppression of the vascular system. When carried to a large extent, they are almost always essentially hurtful; whereas stimulant medicines may often be employed with most advantageous results."

*Rostan* has also strongly expressed a similar opinion in these terse words, *il faut etre avare du sang des vieillards;* and in another passage he says: "the cure of cerebral hæmorrhage must depend on the absorption of the coagulum. Now, in order that this may take place readily, the body must have a certain degree of force or energy. As this force is necessarily feeble in old age, how can the absorption take place, if the system be still further reduced by debilitating measures?"

*Piorry* likewise writes thus: "Drastic purgatives diminish the quantity of the

* The English reader will object to part of the reasoning in this extract; still there is some practical truth in the remarks.—*Rev.*

circulating fluids. They have this advantage over blood-letting, that they drain off the serosity of the blood, without affecting its fibrinous portion."

*Laennec* has quoted from *Corvisart* a case which is strongly confirmatory of the good effects of drastic purgatives in some extreme cases of diseased heart, accompanied with dropsical effusions.

A man, whose speedy death *Corvisart* considered inevitable from such a state of complicated mischief, and who had not obtained any relief from blood-letting or the use of diuretics and various other medicines, applied to a charlatan who had acquired quite a reputation, in the faubourgs of Paris, for the cure of dropsy. This man gave the patient a strong drastic powder, mixed up in two ounces of brandy: it acted upwards of twenty times on the bowels. A similar dose was repeated every day for a week. Under this regimen, the serous diathesis was completely overcome, and the patient lived for ten years afterwards in a very tolerable state of health.

The *great* scope of Dr. *Jourdain's* paper is to recommend the use of drastic purges and also of emetics in the treatment of almost every form of apoplexy, when the constitution is not too much enfeebled. In some cases the employment of stimulants, he says, especially of ammonia, and of depurative bitters, such as *polygala, trifolium aquaticum, marrubium,* &c. along with strong purgatives, is productive of the most beneficial effects. By such treatment a powerful derivative action on the bowels is produced, and the absorbent system is thus stimulated to remove the effused fluid. *Abercrombie* has justly remarked that the first amendment in a case of apoplexy is usually obtained by the action of energetic purgatives; and the same distinguished author—not to mention others, as *Sydenham, Boerhaave, Lieutaud,* &c.—approves of the use of emetics. *Moulin* regarded this class of medicines as so very useful that he proposed the injection of them into the veins, if vomiting could not be induced in the ordinary way. On this proposal, *Abercrombie* remarks that, "this practice must be founded in observation and experience." Dr. Jourdain recommends that they should be repeated at intervals of twelve hours or so. The doses should be large and efficient.

He very judiciously observes,

"Before having recourse to any evacuants in the treatment of apoplexy, ice should be laid upon the head, and at the same time blisters on the arms and sinapisms on the legs."

He again alludes to the good effects of free doses of ammonia, in those cases of cerebral congestion which are brought on by mental emotions or by indigestion. But when the disease is at all connected with obstruction to the return of the venous blood, this, as well as all other stimulants, should be avoided.

*Portal* has observed that "several patients, who have consulted me, have been preserved from apoplectic attacks by the use of sudorific decoctions, sharpened by the addition of liquid ammonia."

Such practice, with the occasional use of emetics, is far preferable to the use of strychnine and other such poisonous substances, in the treatment of palsy.—*Bulletin Medical Belge.*

---

### M. Trousseau on the Use of Blisters.

There is so much practical and *English-like,* so to speak, spirit in a recent letter addressed by M. *Trousseau* of Paris to M. *Brettonneau* of Tours, his former preceptor, that we shall extract several passages, and give a short abstract of the rest.

After alluding to the admirable instructions contained in the chapter of *Stoll's* Ratio Medica, *De quibusdam magni momenti minutiis,* which M. *Bretonneau* had so earnestly recommended to his notice during his pupilage, M. *Trousseau* proceeds :—

"I early took, at your school, antipathy to all empty speculations which lead to no practical end, and a great contempt for the laborious and sterile lucubrations of those who seek for posthumous fame by the discovery of some new auscultatory râle, or by the microscopic detection of some hitherto unnoticed morbid production. *En revanche,* I have always esteemed in a very particular manner such physicians as are willing to descend from the high region of what now-a-days is called *science,* and who will talk with me of what they have found to be really useful in the treatment of any disease, however simple and seemingly trifling the remedy may be. To tell the truth, I have much greater pleasure in meeting with a man who will teach me the best mode of making a poultice than with him who professes to instruct me in the differences between the *râle ronflant* and the *râle sonore,* or how to distinguish the *râle sibilant* from the *soufflant râle,* or this latter from the *turturin râle,* or this again from the *roucoulant râle,* or the *caverneux* from the *cavernuleux râle,* and all such petty distinctions. You indeed in the provinces are sadly *arrierés,* not recognising, I am told, more than the four or five different *râles* described by *Laennec.*"

M. *Trousseau* then good-humouredly confesses that, although he is Professor of Therapeutics to the Faculty of Medicine in Paris, he is often at a loss as to the proper method of performing some of the simplest niceties in practice, such as the dressing of a blister, the application of leeches, &c.

The blistering plaster which M. *Trousseau* prefers to all others, and is the one which has always been used by M. *Bretonneau,* is prepared by adding powdered cantharides to sweet oil, till the mixture acquires the consistence of an electuary. It is then to be nicely spread upon adhesive plaster, and may then be applied to the skin. The interposition of a piece of blotting-paper between the skin and the blister renders the application altogether more cleanly, and diminishes the risk of urinary irritation, without impeding the efficacy of the remedy.

This is the form of blister which MM. *Velpeau* and *Trousseau* have introduced from the hospital at Tours into those at Paris under the name of *vesicatoire Brettonneau,* and is infinitely superior to the numerous preparations which are vended by the French pharmacopolists under the names of *vesicatoires Anglais, vesicatoires par incorporation,* &c.

The vesicant effects of the cantharides plaster seem to be rendered more certain, if its surface is smeared over with the *huile de cantharides par ether*—for which there is the following formula in the last codex :—

Take of powdered cantharides 2 livr.
Sulphuric æther, a sufficient quantity.

Make a tincture by lixiviating the mixture, and draw over the ether by slow distillation. A green thickish oil, which has very strong vesicant properties, is thus obtained.

M. *Trousseau* has of late tried this *etherial extract of cantharides* as a vesicant by itself in the following manner.

A portion of paper (such as is used by women for curling their hair), of the size of the wished-for blister, is to be laid upon a piece of diachylon plaster somewhat larger. A few drops of the *extract* are then to be poured upon it, so as to moisten it sufficiently, without however wetting it. This application causes heat in the part in about two hours, and, in the course of seven, a blister is usually raised. Perhaps the medium time required is from eight to nine hours.

The extreme convenience and cleanliness of this kind of blister must certainly recommend it under many circumstances ; and, as it is not more expensive than the blisters in common use, the facility of the preparation renders it well adapted to military and hospital practice. M. *Bouillaud* employs this style of blister almost constantly at the Hôpital de la Charité ; and already the *ethereal extract* is in great demand at many other of the Paris hospitals.

The extract is of a green colour and of the consistence of olive oil. It deposits a matter of a buttery consistence, which is excessively acrid and irritating. If incautiously applied to the skin, it produces a painful ulceration, which is often very difficult to heal. This deposit is readily soluble in oil, and the solution, if not too weak, is found to be an energetic vesicant.

M. *Trousseau* suggests that, as the ethereal extract seems to contain all the active properties of cantharides, a convenient and useful mode of preparing the tincture might be by dissolving a certain quantity of it in alcohol.—*Journal des Connoissances Medico-Chirurgicales.*

---

### On the Therapeutic Properties of Prussic Acid.

The rapidly fatal effects of even a small dose of this most potent poison are well known to every one. Even in the hands of medical men it has unfortunately, in some cases, produced lamentable effects.

In 1831, at the Bicetre Hospital, one of the most distinguished physicians of that establishment, who had on several occasions successfully prescribed the *sirop cyanique* prepared according to *Majendie's* formula, ordered to seven epileptic patients at the same time a certain and regulated dose of this syrup; but, in place of *Majendie's* preparation being used, most unfortunately that ordered by the Codex was employed—the latter containing a *tenth* part of the pure acid, whereas in the former there is only a hundred and twenty-ninth part. In each dose nearly five grains and a half of the concentrated acid were administered. All the patients died in from twenty minutes to three quarters of an hour after the exhibition of the medicine. On one occasion M. *Trousseau,* whose boldness in the use of certain medicines every one knows and many condemn, exhibited to a hydrophobic patient thirty-six drops of the medicinal acid in one dose. In a few seconds the man fell as if stricken dead: when he recovered, six drops more were given. As soon as the acid this time touched his tongue, he again seemed as if he had been struck with a thunder-bolt. A few minutes afterwards, however, he regained his senses.

The following memoranda on the effects of moderate doses of this medicine have been drawn from the clinical practice of M. *Andral,* whom no one will suspect of an unscrupulous and improper boldness in experimenting on his patients.

*Epilepsy.*—A young soldier, who had most miraculously escaped from being decapitated by three Bedouins near to Constantine, was suddenly seized with frightful convulsions when he reached the nearest sentinel station at the outposts. From that time to the present he has been subject to returns of the fits, at longer or shorter intervals. The attack consists in a paroxysm of violent convulsive movements of almost every part of the body, accompanied with occasional shouts or screams, and a few broken unintelligible words. After a short time, the muscular agitation ceases, and then there supervenes a state of delirium; during which the patient almost invariably reverts to the horrors of his threatened death, at the moment when he escaped. According to his own account, every circumstance of the horrible picture is always vividly presented to his mind during the delirious wandering.

M. *Andral* put him on a course of the hydrocyanic acid, beginning with four, and gradually increasing the dose to eighteen drops—which quantity is equal to three drops of the anhydrous acid. The only symptom produced by the small doses was a sensation of painful heat in the stomach after each dose: this became much more severe as the quantity of the acid was increased, and it was then accompanied with a generally-diffused heat over the entire surface of the body, with vertigo, and a sort of muscular anxiety which made the patient dread the return of the convulsions, These accidents continued for only a few minutes, and passed away insensibly under a gentle perspiration. The dose of the acid was nevertheless increased to twenty drops. The burning sensation in the stomach was described by the patient to irradiate to the chest, limbs, and even to the very points of the toes and fingers. On one occasion, after experiencing dizziness, he fell down as if struck by a blow upon the head, and remained in a state of delirium for nearly two hours. The progress of this case was altogether unsatisfactory; for the tendency to delirium seemed to become greater, and nothing occurred which gave any encouragement to the hope of effecting a cure.*

From the results of this and of many other cases of convulsive disease, we are led to regard the prussic acid as a very inefficient remedy. The diseases in which it has been found most useful are various affections of the stomach and also of the lungs and heart. In cardialgia and morbid irritability of the stomach, in asthma and pertussis, and in hypertrophy of the heart, decidedly good effects have been derived from its administration. One of the best formulæ for its exhibition is in almond emulsion, or in some mild bitter infusion, with or without the addition of digitalis. It may also be usefully added to the common effervescing mixture, which is so useful a vehicle for the administration of many active medicines.

As an external application, the hydrocyanic acid is certainly very beneficial in various irritated or inflamed conditions of the skin. Usually it is best to add it to the common Goulard lotion in the proportion of from ʒij.—iv. to half a pint, and this should be applied lukewarm to the part, which should then be covered with a piece of oil-silk. In this simple manner a constant moisture is kept up.—*Bulletin Medical.*

---

### STATISTICAL RESEARCHES ON THE DURATION OF LIFE.

In one of the recent numbers of this Review we gave an abstract of the first part of M. *Bellefroid's* researches on this curious subject. We now proceed to notice the second part, and extract what seems to us the most interesting particulars.

### *Influence of Profession on the Duration of Life.*

Without making any prefatory remarks, we present the following table as conveying the results of Dr. *Bellefroid's* calculations, founded upon the data of numerous registries.

Calculated from the 28th year of age, the probability of life may be estimated in—

| | |
|---|---|
| Catholic priests to be to the age of .... | 69 years. |
| Protestant clergymen ............... | 65 |
| Savans ........................... | 66 |
| Professors in Universities ............. | 63½ |
| Military Officers ................... | 64½ |
| Poets............................ | 61 |
| Artists ...................... ..... | 62 |

---

* In our opinion, prussic acid is not the medicine from which we should have expected much benefit in the treatment of such a case. The use of carbonate of iron or of some other ferruginous preparation, and of free doses of hyosciamus at bed-time, might have been productive of decided benefit; the object being to tranquillize the nervous system, and to invigorate the general health. Cold shower bathing also is a useful remedy in such cases.—*Rev.*

| | |
|---|---|
| Farmers | 64 years. |
| Merchants | 64 |
| Physicians—theoretical | 64 |
| Physicians—practical | 60 |
| Female Artists, Literary Characters, Actresses, &c. | 63½ |
| Barristers | 62 |
| Kings | 56½ |
| Mendicants | 56 |

As an appendage to the preceding table, we may state that it has been estimated that 13 only out of a hundred kings attain the age of seventy years; whereas to the same period of life not fewer than 46 catholic priests, 41 savans, 37 farmers, 36 military officers, 33 professors, 29 barristers, 26 physicians, and 21 mendicants, are calculated to survive; the date, from which the calculation is taken, being usually the 21st or 22d years of life.

The general conclusions to be derived from these data must be at once obvious. The average life of kings is short, compared with that of clergymen and philosophers, who—

Along the cool sequestered vale of life,
Have kept the noiseless tenour of their way.

And who can wonder at this, when they consider the career of the former; their life being one of ceaseless and most fatiguing excitement; minds as well as bodies inflamed by ever-recurring sensual enjoyment, and exhausted by the constant turmoil of unrestrained passions. Tossed about between the extremes of joy and grief, it is only by stealth, as it were, that they can taste that peace of heart and that silence of the feelings which are so essential to the calm and unconscious flow of life. How different the career of the contented Catholic Priest! The temperance, the chastity, the absence of all the violent passions, associated too with a due activity alike of the body and of the mind—these are the elements of the healthfulness and longevity of such a profession.

If we now turn to the life of poets and artists, we find that on the whole they too do not live so long as clergymen: the excitability of their passions, and the over-vivacity of their temperament being doubtless unfavourable to the tranquil movements of prolonged health.

We may be at first surprised to hear that military officers are among the most favoured of the classes, and also that the life of barristers is not so good as that of artists and poets. But we should remember that the above table comprises only the superior officers, who are almost always 30 years of age or upwards when they attain to high rank in either service; and that many barristers are not more than 20 years of age, and scarcely any exceed the age of 25, when they become so.

So much for other professions: let us now hear what our author has to say on the case of *doctors.*

"Their profession," he remarks, "does not make any great demands on the imagination; and moreover the mental effort expended upon its pursuit has no tendency to exhaust the mind. With medical men it is rather the machine, the body, than its moving principle, the soul, which wears itself out: *aliis inserviendo consumuntur, aliis medendo moriuntur.* The physician is exposed to all sorts of revolutions of his material life; his sleep is apt to be disturbed, his meals to be interrupted; his sensibility is often wounded by a constant spectacle of human suffering and distress; his life, in short, is a ceaseless travel through hot and cold, dry and wet; and at the end of all his anxieties and fatigues, his recompence is too often a miserable fee; and if it is sometimes given with marks of gratitude, this is quickly forgotten when health is restored!

*Das ist ein Leben, das heisst ein Leben!*"

From the preceding memoranda the reader will doubtless agree with our author, that he who desires long life, should pray to be neither a king nor a beggar;* "to have neither poverty nor riches;" not a pampering superfluity on the one hand, nor a pinched insufficiency on the other; and withal have not too much pity, nor too lively an imagination; lastly, *he should not be a physician.*

"This prescription," adds our author, "is infallible."

*Influence of Ease and of Poverty on the Duration of Life.*

The following brief table drawn up by M. *Benoiston de Chateauneuf* is more impressive on this subject than the most elaborate disquisition. According to his researches the mortality per cent. among the rich and poor may be estimated as follows:—

| Age. | Rich. | Poor. |
|---|---|---|
| From 25 to 40 years .... | 2,05 .... | 5,50 |
| 40 to 50 years .... | 2,44 .... | 4,26 |
| 50 to 60 years .... | 3,49 .... | 7,18 |
| 60 to 70 years .... | 7,37 .... | 15,01 |
| 70 to 80 years .... | 14,89 .... | 28,73 |
| 80 to 90 years .... | 27,87 ... | 0,00 |

It has been calculated that in Paris there does not survive one pauper person beyond 80 years of age; whereas, among the richer classes, there are not a few octogenarians.

Our author sums up his remarks with the following conclusions:—

1. That poverty exercises a directly injurious influence on the duration of life.
2. That at least 28 per cent. among the poor die from this cause before the fifth year of life, and even more than this proportion where fecundity exists among a population.
3. That however, when all other circumstances are alike, there is a rather smaller mortality among the poor than among the rich between the 20th and the 30th year of life.†

Lastly, that fewer die from poverty in an agricultural than in a manufacturing population.

We now proceed to another topic:—

*Influence of Climate on the Duration of Life.*

There has been considerable difference of opinion on this point among statistical writers. While *Casper* and *Süsmilch* assert that it is neither the climate nor the soil of the country which is inhabited, but only man himself considered as a collective being, that regulates the duration of life, M. *Moreau de Jonnés*—(a French academician who, our author says, has greatly *abused* statistics)—has distinctly declared that a cold and even a rigorous climate, especially in the neighbourhood of the sea, is directly favourable to the prolongation of life.

Let us briefly endeavour to ascertain how far ascertained facts bear out this assertion.

The mortality among the Icelanders has been estimated at one in every thirty-three of the inhabitants—a proportion which is somewhat less than in some districts of France and Spain, such as in the departments of Vaucluse and the Eastern Pyrenees, and very considerably less than that among the Russian-Greek

* Our author, in another part of his paper, remarks, that the average life of mendicants, if taken at 25 years of age, is nearly 55 years; whereas that of the kings of France has not exceeded 48 or 49 years, and it is almost as long as that of German physicians and of English poets.

† Our author attributes this somewhat unexpected result of his enquiries to the pernicious effects which flow from the facilities which the rich have of gratifying their passions, during the epoch when these are most vehement.

population. It exceeds only by one or two per cent. the average mortality in the Prussian dominions, which would seem to be actually greater than that among the slave population of Havannah. But then the average duration of life in England, France and Belgium is considerably higher than in Iceland—by at least 10 years in the first, and 7 years in the latter two countries.

Again, to shew how little the mere influence of a cold climate, apart from other circumstances, can be deemed favourable to the prolongation of life, we may state that, whereas there is a mortality of one in every 25.8 of the Russian-Greek population, and the average duration of life does not exceed 21 or 22 years, the mortality in some districts of Switzerland, where the climate is quite as severe as in Russia, does not exceed one in every 49 of the inhabitants, and the average duration of life is nearly as high as 49 years—a ratio which exceeds the former by at least a double.

Then let us take an instance of two climates, strikingly opposed to each other—viz. humid and marshy Holland on the one hand, and sandy Brandenburg on the other. And yet, if we examine the bills of mortality in these two countries, we shall find that they exhibit a very marked approximation, the one to the other; as appears from the following table published in the inaugural dissertation by Casper and Wiskott.

The average duration of life is—

| At the age of | In Holland, | In Branderburg, |
|---|---|---|
| 0 years | 34,5 years | 30, years. |
| 5 „ | 44,4 „ | 44,3 „ |
| 10 „ | 42,6 „ | 42,8 „ |
| 20 „ | 36,2 „ | 35,6 „ |
| 50 „ | 19,4 „ | 17,7 „ |
| 70 „ | 9,1 „ | 8,1 „ |

From these and similar data we may reasonably conclude, that temperate climates are most favourable to the duration of life, but that those, the rigour of which is excessive, may be to a certain extent overcome by human intelligence.

We do not however assent to the opinion of *Casper* and *Sussmilch* as to the utter nullity of the influence of climate; but, as to that of *M. Moreau de Jonnés*, it is utterly untenable. The error of the latter gentleman has probably arisen from his having confounded the general or average duration of life with the mere ratio or frequency of extreme longevity. Instances of this—extreme longevity—are more frequently met with in hot and in cold than in temperate climates. Every one may have heard of the number of very aged people among the inhabitants of Iceland, and some other cold islands. But it does not necessarily follow that the average duration of life is unusually prolonged.

### *Has the Duration of Life increased?*

This is a question which is of no easy solution, in consequence of our almost entire want of accurate information on this subject in remote times. There are indeed two historical fragments, which may, in some measure at least, assist us in this enquiry. The first is from the writings of *Herodotus*, who, in treating of the kings of Egypt, says, "three hundred generations make six thousand years; for three generations are equivalent to one hundred years." Although it is by no means certain whether this expression is meant to apply to the generations of the kings alone, or to those of the Egyptian people generally, or to those of the Greeks, we must acknowledge that the figure mentioned by the old historian agrees very nearly with the medium duration of generations in modern times. M. *Villot* has estimated this in the case of the male population of Paris at 33⅓ years, and M. *Quetelet* at 40 years in the case of both sexes in Belgium. (This latter estimate is acknowledged to be considerably too high).

The second fragment is from the works of *Ulpian*, who lived at the beginning of the third century.

He gives a table of the probability of life among the Romans, taken at different ages, for the purpose of making certain calculations. But its results being so widely different from those of any well-authenticated tables, it must certainly be confessed that no importance can possibly be attached to them.

According to the researches of *Marshall,* it would seem that the probable life among the inhabitants of London has increased by about seven years within the last century, and the average life, during the same time, by about five years: the latter being, in 1728—39, 25 years; and in 1820—29, 30½ years.

A still greater augmentation than this seems to have taken place among the inhabitants of Geneva, where life has gained, it has been calculated, nearly 40 years in the course of two centuries. This very rapid increase is however doubtless attributable, in part at least, to the cessation of the plagues and famines which used frequently to devastate this republic.

At Berlin also, the value of life has, it is calculated, increased by 4½ years in the course of half a century.

We must not forget to mention here the important discoveries of Mr. *Finlayson* on this subject.

This gentleman has found that the mortality throughout England has singularly diminished during the past century; but that this diminution has not been equal at all the epochs of life, nor to the same extent in the two sexes: for, while the mortality among youths and adult men seems to have remained nearly stationary for the last fifty years, that among girls and women has decidedly decreased.

Mr. Finlayson has also compared with great care the average duration of life in persons who had insured during the 17th and during the 18th centuries, and he has found that in the course of 96 years there has been a gain of

| | | | | |
|---|---|---|---|---|
| 10 years | .... | at the | age of | 5 years. |
| 9 — | .... | — | — | 10 — |
| 9 — | .... | — | — | 20 — |
| 8½ — | .... | — | — | 30 — |
| 7 — | .... | — | — | 40 — |
| 5 — | .... | — | — | 50 — |
| 3 — | .... | — | — | 60 — |

These results shew that the easy and affluent classes in England have during the last century made immense progress in what the Greeks would call *Macrobiotechnie.*

This progress however, has been, as indeed might be expected, much greater among the inhabitants of large towns than among the residents in the country. We may also add here, that the average duration of life in the whole human race will probably never exceed 33 years or thereabouts, and that it will need many ages of progress still to come, before even this ratio is attained.

Before concluding our remarks, we shall add a word or two on some of the causes which have probably operated in enhancing the value of life in modern times. Not to dwell upon the comparative infrequency of pestilential maladies, the *partial*—would that we were not obliged to use this epithet—subjugation of the small-pox, the superior cleanliness of our large towns, the numerous modes and appliances of relief to the destitute and sick, it may be reasonably believed that the improved condition of the healing art has contributed somewhat to the prolongation of human life.—*Bulletin Medical Belge.*

### On the Treatment of Encysted Ganglionic Swellings.

M. *Baudens*, one of the leading military surgeons in France, prefaces some remarks on the treatment of such tumors, by alluding to the remarkable occasional increase and decrease in the frequency of certain diseases, which cannot be supposed to be at all dependent upon atmospheric vicissitudes.

"There are," says he, "some maladies which seem to disappear entirely from the nosological catalogue for three or six months at a time, and again to make their appearance in groupes of four, five, or more at a time, without our being able to assign to such phases any epidemic cause. We may account for the occasional prevalence of such diseases as erysipelas, typhus, small-pox and so forth; but no condition of weather can well be supposed to influence the frequency of *fractures, sprains, hydroceles, sarcoceles*, &c. such as during the last two years we have observed in this hospital, (at Lille)—at longer or shorter intervals of time, and sometimes in the number of five or six on one day of clinical admittance." This remark holds true of encysted swellings also, aneurysms, and many other surgical disorders.

M. *Baudens* then gives the following description of the mode of development of ganglionic swellings:—

"If the sheath of a tendon, which has been subjected to strong muscular efforts or to any prolonged compression, happens to give way at one point of its circumference, either spontaneously or from ulceration or from some other cause, the synovia escapes into the surrounding cellular texture, and a swelling, not unlike to an incipient aneurysm, follows; and, according as there is a rupture or only a mere dilatation of the enveloping tissues, so is the swelling either circumscribed or diffused. It is more than probable that the bursa is in almost all cases merely dilated, and not ruptured; as we know that the most successful practice in dispersing ganglionic swellings is to cause their contents to be effused into the adjoining cellular tissue. These contents, while still within the cyst, sometimes undergo considerable changes; by either becoming thicker, and gradually acquiring a steatomatous consistence, or by hydatidiform nodules being slowly developed in the fluid. The latter change is often indicated by the tumor acquiring a peculiar crackling sensation on being squeezed between the finger, or when pressed on: this arises from the rubbing of these nodules, the one upon the other.

*Treatment.*—If ganglionic swellings form quickly, and especially if they be attended with pain and uneasiness, the application in the first instance of a few leeches, and subsequently of mercurial ointment, may suffice to dissipate them.

Of late years, M. *Baudens* has succeeded in dispersing the synovial tumor in almost every instance, when the disease has been more chronic, and has resisted these measures, by having recourse to acupuncturation, in such a manner that the contents become diffused in the surrounding cellular texture. When the contents of the sac are too thick to permit its ready escape in this manner, we should then use in place of the needle a cataract knife, which may be introduced readily under the skin, and with which the walls of the cyst may be freely divided in numerous directions. The admission of air into the interior of the sac is thus altogether prevented. The part should then be immersed in cold water for several hours, and firm compression be afterwards kept up by means of compresses and bandage.

This practice is very much preferable to the incision, or the extraction, of ganglionic swelling—the former of these methods being not unfrequently followed by most troublesome symptoms, and the latter being very unnecessarily severe. It is of great consequence to avoid the risk of severe inflammation attacking the tendons on which the ganglions have formed; as their free movements are after such attacks almost always most seriously injured. When the ganglion forms under the transverse ligament of the carpus, it is sometimes necessary to divide this across, before a cure can be effected. Before however having recourse to this operation, we should give a fair trial to the gentler one of opening the sac freely under the integuments with a cataract-knife, as recommended above.

M. *Baudens* has repeatedly adopted the practice of rupturing ganglionic tumors by firmly squeezing these between the fingers, or striking them smartly with a book. He prefers, however, the plan of puncturing them with a needle, or with a small cataract-knife.—*La Lançette Française.*

---

### CASE OF MILKY URINE, WITH ITS CHEMICAL ANALYSIS.

A lady, born in the Isle of France, observed in her twenty-fifth year, for the first time, that her urine was remarkably milky. This peculiar state continued for nearly three weeks, during which her general strength diminished very sensibly. From this period of life to the age of seventy-five, the urine was every now and then of a milky aspect, or glairous and viscid, occasionally becoming almost gelatinous, and not unfrequently having a good deal of blood mixed with it. Whenever this unnatural condition of the secretion was present, her strength and appetite decayed. When seventy-six years old, she left the Isle of Bourbon and visited France. For a twelvemonth previously, the urine had been natural, and her general health good. During the voyage to Europe, however, the water became thicker and whiter than it had ever been before, and her strength was much reduced. When she reached Paris, she had several inflammatory attacks on the chest. During these it was always observed that the urine was clear while the diet was low, and became milky, glairous and blended with blood, when the patient took any nourishing food.

From this time, her health became much more enfeebled, and the system was seldom or never free from a feverish irritation. Bark, steel, and various astringents were administered; but not with any success. More benefit was obtained from morphia than from any other remedy.

M. *Quevenne*, who carefully analysed the urine, has appended the following conclusions to the account of his experiments.

1. The urine exhibited none of the characters of milk except its whitish aspect; and, when examined with the microscope, no traces of fatty globules or of caseum were discoverable.

2. The white appearance of the urine in the present case cannot be attributed to the admixture of purulent matter, as the microscope displayed only a very few globules which could possibly be taken for those of genuine pus; and moreover the urine, when allowed to stand, remained quite opaque and homogeneous—which would not have been the case if there were any purulent admixture with it.

3. If we consider that the urine in this case exhibited under the microscope sanguineous globules, and also that the fatty matter blended with it was in a peculiar state of suspension or combination not recognisable by the microscope—a condition to which we find an analogous one only in the chyle—we are naturally led to the idea that this fluid, the chyle, had probably found its way into and become blended with the urine. In this way we can account for the superabundant secretion of fatty matter, and for the marked diminution in the proportion of the usual salts found in healthy urine.

These analytical conclusions agree well with the numerous observations reported by M. *Rayer* in his Memoir on the *Hæmaturia* of the Isle of France, and also with the experimental remarks of M. *Guibourt* on the case related in the Journal *l'Experience* for May, 1830. (Vide Medico-Chirurgical Review, July, 1839.)—*Journal des Connoiss. Medicales.*

